# NETest® 2.0—A decade of innovation in neuroendocrine tumor diagnostics

**DOI:** 10.1111/jne.70002

**Published:** 2025-02-13

**Authors:** M. Kidd, I. A. Drozdov, A. Chirindel, G. Nicolas, D. Imagawa, A. Gulati, T. Tsuchikawa, V. Prasad, A. B. Halim, J. Strosberg

**Affiliations:** ^1^ Wren Laboratories Branford Connecticut USA; ^2^ Bering Research London UK; ^3^ University of Basel Basel Switzerland; ^4^ University of California—Irvine Orange California USA; ^5^ Bennett Cancer Center Stamford Connecticut USA; ^6^ Hokkaido University Hospital Sapporo Japan; ^7^ Mallinckrodt Institute of Radiology Washington University in St. Louis St. Louis Missouri USA; ^8^ Moffitt Cancer Center Tampa Florida USA

**Keywords:** biomarker, diagnostic accuracy, NETest, neuroendocrine tumor, qPCR

## Abstract

Gastroenteropancreatic neuroendocrine neoplasms (GEP‐NENs) are challenging to diagnose and manage. Because there is a critical need for a reliable biomarker, we previously developed the NETest, a liquid biopsy test that quantifies the expression of 51 neuroendocrine tumor (NET)‐specific genes in blood using real‐time PCR (NETest 1.0). In this study, we have leveraged our well‐established laboratory approach (blood collection, RNA isolation, qPCR) with contemporary supervised machine learning methods and expanded training and testing sets to improve the discrimination and calibration of the NETest algorithm (NETest 2.0). qPCR measurements of RNA‐stabilized blood‐derived gene expression of 51 NET markers were used to train two supervised classifiers. The first classifier trained on 78 Controls and 162 NETs, distinguishing NETs from controls; the second, trained on 134 stable disease samples, 61 progressive disease samples, differentiated stable from progressive NET disease. In all cases, 80% of data was retained for model training, while remaining 20% were used for performance evaluation. The predictive performance of the AI system was assessed using sensitivity, specificity, and Area under Received Operating Characteristic curves (AUROC). The algorithm with the highest performance was retained for validation in two independent validation sets. Validation Cohort #I consisted of 277 patients and 186 healthy controls from the United States, Latin America, Europe, Africa and Asia, while Validation Cohort #II comprised 291 European patients from the Swiss NET Registry. A specificity cohort of 147 gastrointestinal, pancreatic and lung malignancies (non‐NETs) was also evaluated. NETest 2.0 Algorithm #1 (Random Forest/gene expression normalized to *ATG4B*) achieved an AUROC of 0.91 for distinguishing NETs from controls (Validation Cohort #I), with a sensitivity of 95% and specificity of 81%. In Validation Cohort #II, 92% of NETs with image‐positive disease were detected. The AUROC for differentiating NETs from other malignancies was 0.95; the sensitivity was 92% and specificity 90%. NETest 2.0 Algorithm #2 (Random Forest/gene expression normalized to *ALG9*) demonstrated an AUROC of 0.81 in Validation Cohort #I and 0.82 in Validation Cohort #II for differentiating stable from progressive disease, with specificities of 81% and 82%, respectively. Model performance was not affected by gender, ethnicity or age. Substantial improvements in performance for both algorithms were identified in head‐to‐head comparisons with NETest 1.0 (diagnostic: *p* = 1.73 × 10^−9^; prognostic: *p* = 1.02 × 10^−10^). NETest 2.0 exhibited improved diagnostic and prognostic capabilities over NETest 1.0. The assay also demonstrated improved sensitivity for differentiating NETs from other gastrointestinal, pancreatic and lung malignancies. The validation of this tool in geographically diverse cohorts highlights their potential for widespread clinical use.

## 1 INTRODUCTION

Gastroenteropancreatic neuroendocrine neoplasms, once considered rare, are now recognized as relatively common, with an incidence of 6.8 per 100,000, comparable to testicular tumors, Hodgkin's disease, gliomas and multiple myeloma.^1^ Their prevalence is estimated at 35 per 100,000.^1^ These neoplasms pose a significant challenge, since 50%–70% are metastatic at diagnosis. Thus, sensitive and robust biomarkers for diagnosis, disease progression assessment, and treatment efficacy monitoring are crucial to patient management.^2^

The Identification of peripherally accessible molecular fingerprints using PCR amplification of target genes has been successful in other cancers, such as breast, colon and prostate cancers.^3^, ^4^, ^5^ Previously, we reported the development of a neuroendocrine neoplasia‐associated circulating signature (the NETest®) for NETs.^6^ This is a liquid biopsy assay that measures the expression of 51 NET‐related genes in blood using real‐time PCR. The NETest is a standardized, reproducible, CLIA/CAP‐regulated clinical assay that has been independently validated as a diagnostic for NETs.^7^, ^8^ This molecular approach has demonstrated greater sensitivity than measuring secretory products, for example, Chromogranin A, for NET disease detection and management.^7^, ^9^

In the assay, RNA is extracted from blood, converted to cDNA, and gene expression quantified using qPCR on primer spotted plates following CAP‐ and CLIA‐validated protocols.^10^ Results are normalized and compared to a cohort of known controls and NETs using algorithms (Support Vector Machines, LDA, KNN and Bayes) which are not always scale‐insensitive. The assay could be used to distinguish whether the sample was a NET or a control.^6^ The score is augmented with normalized expression of genes reflecting tumor behavior (proliferation, metabolism, secretion, epigenetics, and pluripotency) and is scaled from 0% to100%.^11^ Disease activity is categorized into normal (≤20), low (21–40), intermediate (41–79) and high (80–100).^11^

The initial algorithm and step‐wise scoring system (NETest 1.0) was developed in 2015 and is based on a relatively small number of blood samples (57 controls/63 NETs) collected in EDTA tubes.^11^ False positives (~30%) have been identified for other gastrointestinal (GI) malignancies^8^, ^9^ and lung diseases including non‐NET neoplasia's and interstitial pulmonary fibrosis (IPF) can also have positive scores.^12^, ^13^ Consecutive scores can fluctuate which has raised questions regarding the source of variability.^14^ Collection protocols have subsequently evolved to now directly stabilize mRNA, for example, PaxGene tubes. Such stabilization will affect mRNA levels and may alter algorithmic outputs (that are based on gene expression) and assay results. Classifiers and algorithms have also significantly evolved to become more accurate through more rigorous training approaches as well as by minimizing issues associated with outliers and data scale/distance calculations.^15^, ^16^ Continuous scores are now known to allow for the identification of a natural cut‐off for binary decision making; they are also typically well‐calibrated irrespective of the diversity and scale of data.^17^

We recently developed and validated an algorithm for detecting prostate cancer‐associated gene expression in non‐EDTA collected RNA‐stabilized blood, which exhibits high model discrimination and calibration and provides for stable consecutive scores.^5^ In the current study, we report on the development of updated NET algorithms (NETest 2.0) from RNA‐stabilized blood samples. We undertook this to ensure that the NETest could provide the most accurate data currently possible. We specifically wanted to develop an assay that was stable and could also differentiate NETs from other malignancies. In the United States, per CLIA/CAP guidelines, biomarker approaches are routinely evaluated, to ensure accuracy and clinical utility. Our goal was to minimize any false negative or positive results or the misclassification of a sample.

We used a larger Derivation Dataset (*n* = 240) compared to the original NETest (*n* = 130), and included two substantially sized, independent Validation sets. Validation set #1 comprised 463 NETs and controls, while Validation set #2 included 291 NET cases from a Swiss Registry study.^18^ Additional specificity cohorts including other malignancies (*n* = 147), subjects with small cell lung cancer (SCLC) and individuals with IPF (*n* = 50) were evaluated. This provided the basis for algorithm testing and validation of NETest 2.0. Our aims were to (1) utilize the original NETest PCR assay protocol to optimize two algorithms for NET management: one to differentiate a NET from a control and one to distinguish progressive from stable disease as this is considered prognostic,^19^ and (2) compare performance metrics between NETest 2.0 and the original NETest 1.0 algorithmic output. The differences between these two algorithms are identified in Table 1.

**TABLE 1:** Similarities and differences between NETest 1.0 and NETest 2.0.

	NETest 1.0	NETest 2.0
Collection tube	EDTA	Wren RNA stabilization buffer (EDTA tube and buffer)
Transportation	Dry ice	Ambient
RNA isolation	Qiagen: QIAamp RNA blood MiniKit (no Trizol)	Trizol LS isolation, RNeasy MiniKit (Qiagen)
cDNA synthesis	Life Technologies: High Capacity RT Kit	Life Technologies: High Capacity RT Kit
qPCR protocol	Life Technologies: liquid primers	Life Technologies: pre‐spotted plates (lyophilized primers)
Algorithm	2‐step	2‐step
NET diagnosis	4 classifiers including: SVM LDA KNN Bayes	1 classifier RF
Gene expression input	*ALG9*‐normalized	*ATG4B*‐normalized
Diagnostic output	0–20 = control/non‐NET 21–100 = NET	0–49 = control/non‐NET 50–100 = NET
NET prognosis	Expression from 5 omes Epigenome Metabolome Plurome Proliferome SSTrome	1 classifier RF
Gene expression input	*ALG9*‐normalized	*ALG9*‐normalized
Diagnostic output	21–40 = stable disease 41–79 = intermediate disease 80–100 = progressive disease	0–39 = stable disease 40–100 = progressive disease

## 2 METHODS

### 2.1 Ethics approvals

Ethics approvals were obtained from the Western Institutional Review/WCG Board (WIRB20191743) and the University of Basel (2020‐01339). The study protocol was approved by and conducted in accordance with Good Clinical Practice principles. Informed consent was obtained from all study participants. Blood samples and clinical histories were collected, and all samples along with personal health information were de‐identified. Information including demographic data, current clinical status, treatment, and imaging data was compiled into a database for subsequent evaluation.

### 2.2 Blood samples

**FIGURE 1:**
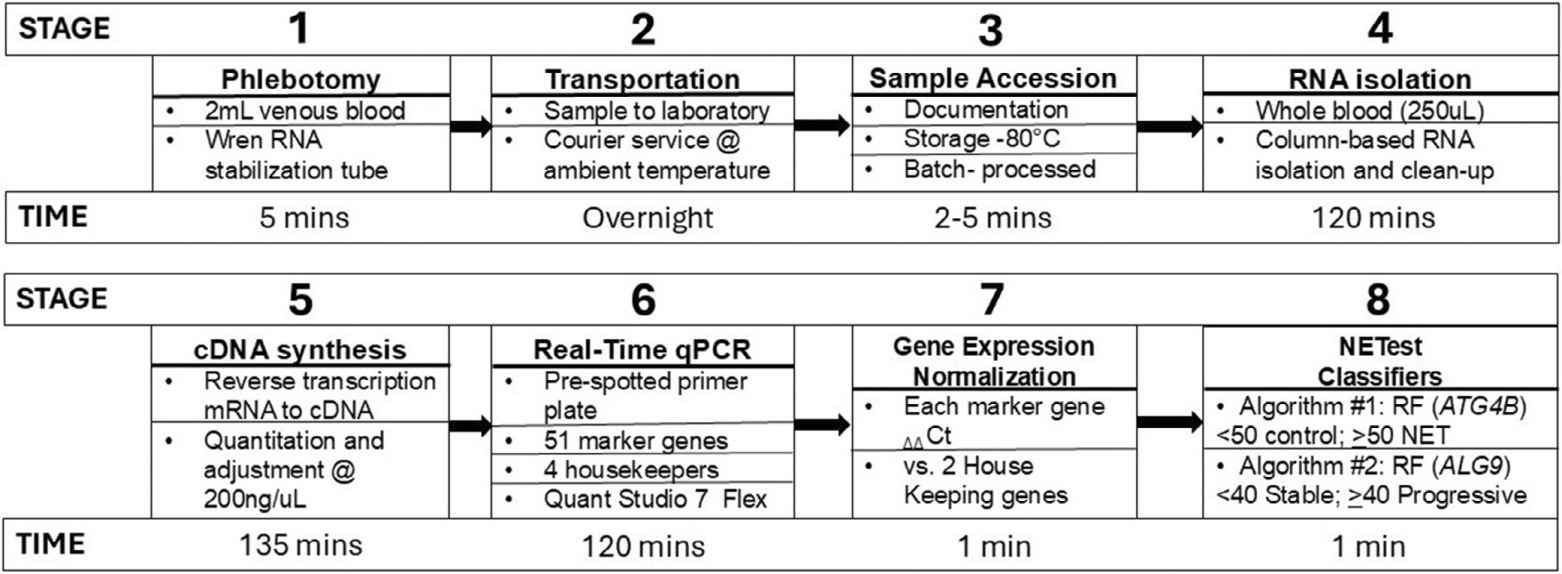
Overview of Wren's NETest workflow. RF = random forest.

Peripheral whole blood samples (2 mL) were collected into 10 mL plastic blood‐collection tubes containing 4 mL of RNA stabilization buffer based on guanidinium hydrochloride as previously described.^20^ Stabilized samples were de‐identified at the source, anonymously coded, and stored at −80°C within 2 h. No pre‐analytic processing was undertaken. All samples were sent to Wren Laboratories LLC for processing and gene expression measurement. Transportation was carried out at ambient temperature by an independent commercial courier (Workflow; Figure 1).

### 2.3 Sample processing

*RNA isolation*: RNA was extracted isolated using the RNEasy MiniKit (Qiagen, Valencia, CA; RNA quality ≥1.8 A_260:280_ ratio, mRNA production: 2.6–34 μg/sample).^21^

*qPCR*: cDNA was generated using the High‐Capacity cDNA Reverse Transcriptase Kit (cDNA production 2000–2500 ng/μL). Real‐time PCR was performed on pre‐spotted TaqMan PCR primer plates using 200 ng/μL of cDNA/well.^10^ A positive control (derived from a 1:1 mix of two NET cells lines, H727 and H720) was included on each plate. Target transcript levels were normalized to four different housekeeping genes: *ALG9*, *ATG4B*, *RHOA*, and *TXNIP*, and quantified using _ΔΔ_C_T_.^21^

### 2.4 Model (NETest 2.0) training

1. Algorithm #1: *Inclusion criteria and sample collection*: The Derivation Dataset for Algorithm #1 (controls vs. NET) comprised 240 subjects some of which had been previously utilized for omic evaluation for the original NETest (2015, 2021).^11^, ^22^ The dataset included 78 controls [median age: 55 (22–80)] and 162 NET patients [median age: 59 (24–92)] (Figure S1). All patients had previously been treated. The clinical status, current treatment and individual demographics are included in Table S1.
  A power analysis identifies the sample size is statistically sufficient for discovery. Assuming a difference in means of 0.95, a Standard Deviation of 2 and a significance level of 0.05, *n* = 71 samples are required per group to achieve 80% power (https://homepage.univie.ac.at/robin.ristl/samplesize.php?test=ttest).
2. *Ground truths*: Ground truths were defined as follows:
  - NET: histologically confirmed diagnosis of neuroendocrine neoplasm.
  NET types included gastroenteropancreatic (stomach, pancreas, small bowel, colon, rectum) and bronchopulmonary (lung) NETs, covering disease stages I‐IV and differentiation status G1‐G3.
3. Healthy controls: Individuals with no current or history of malignancy.
4. *Algorithm #2: Inclusion criteria and sample collection*: The Derivation Dataset for Algorithm #2 comprised 195 subjects. The Derivation Dataset included *n* = 134 with Stable disease [median age: 60 (24–92)], and *n* = 61 with Progressive disease [median age: 61 (25–87)] (Figure S1). All patients had prior treatments. The current clinical and treatment status and individual demographics are included in Table S2.
5. *Ground truths*: Ground truths include the following definitions.
  - Progressive disease: Image‐confirmed evidence of disease progression (CT/MRI and/or ^68^Ga‐PET‐SSA scan, minimum of 2 scans, progression [RECIST‐increase in tumor size or detection of new lesions] within the previous 6 months).
  - Stable disease: Image‐confirmed evidence (minimum of 2 scans, at least 6‐months of progression free survival) of stable disease (CT/MRI and/or ^68^Ga‐PET‐SSA scan).
  Both cohorts included individuals in watch‐and‐wait/surveillance programs and on treatment.
6. *Classifier development—Algorithms #1 and #2 Data partitioning*: Sample gene expression values were randomly stratified into training (80%) and testing (20%) sets, with each strata preserving the ratio of cases to controls. To prevent information leakage between training and testing sets, each split contained unique patient identifiers (Figures S2–S5). *Feature engineering*: Biomarkers (blood gene expression) were supplemented with additional features—“Omes”—calculated as a sum of normalized gene expression values. Omes were derived as published (2021)^22^ and details of individual genes in each of the 14 Omes are included (Table S3): *Model training*: Four supervised classifiers were selected for algorithm training: Random Forest (RF), SVM, Logistic Regression (LR), and Gradient Boosted Trees (GBT). The following hyperparameters were tuned during model cross‐validation runs:
  - SVM: Regularization parameter *C* = [1, 10, 100], Kernel = [linear, radial basis function].
  - LR: Regularization parameter *C* = 20 values, logarithmically distributed between −4 and 4, solver = [lbgfs, newton‐cg, liblinear, sag, saga].
  - GBT: Number of trees = [100, 250, 500, 750, 1000], maximum tree depth = [2, 5, 8, 10, 20].
  - RF: Number of trees = [100, 250, 500, 750, 1000], maximum tree depth = [2, 5, 8, 10, 20].
  The best model hyperparameters were selected using an exhaustive grid search with five‐fold cross‐validation.

### 2.5 NETest 2.0 Algorithm #1 and #2 model validation

*Sample collection and inclusion criteria*: To evaluate whether NETest outputs could reliably differentiate NETs from healthy subjects and individuals with other malignancies or conditions, the algorithm was validated in independent, retrospectively collected testing sets. Two validation cohorts and 3 evaluation cohorts were examined. Sample collection times and cohort age distributions for all 5 cohorts are included in Figure S1. Clinical and treatment status for both NET cohorts are included in the Supporting Information.

#### 2.5.1 Validation cohorts (*n* = 2)

*Cohort #I*: This included 277 US, Latin American and Japanese patients (collected from March 2022 to April 2024) and 186 control blood samples. NETs comprised all individuals undergoing NETest for clinical disease management. All patients exhibited image‐positive disease (IPD), with no exclusion criteria applied (Table S4). The control group consisted of healthy volunteers (Table S4). Disease status was available for 255 NETs, including 192 Stable and 63 Progressive subjects.

*Cohort #II*: This included 291 NET patients as part of a Swiss NET Registry study.^18^ All had a histological diagnosis of a NET, and all had undergone ^68^Ga‐PET imaging (Table S5). Eighty‐nine percent (259/291) had image‐detectable/positive disease (IPD) and 32 (11%) were image‐negative disease (IND). Disease status was defined as for the discovery cohort (discussed above).

#### 2.5.2 Evaluation cohorts (*n* = 3)

*Evaluation Cohort #I*: This included 147 malignancies from different GI sites (*n* = 61), pancreas (*n* = 23), lung (*n* = 59) and others (*n* = 4, including 1x GIST and 3 kidney cancers). Demographics and sites of collection are included in Table S6.

*Evaluation Cohort #II*: This included 19 SCLC. Demographics and sites of collection are included in Table S6.

*Evaluation Cohort #III*: This included 50 IPFs. Demographics and sites of collection are included in Table S6.

### 2.6 Reproducibility and variability evaluations

1. *Intra‐ and inter‐assay variability*: The intra‐ and inter‐assay variability was assessed using 5 different clinical samples. A total of 78 (intra‐assay: paired samples) and 146 inter‐assay PCR runs were undertaken by two different operators on two different QS7 Flex machines, using two different experimental lots over a 20‐day period. C_t_ values (averaged over the 55 genes) were evaluated. Intra‐ and inter‐assay variability was calculated for individual samples, for each of the operators, for each of the two lots and for each of the two machines. These analytical studies were undertaken to meet CLSI guidelines (EP05‐A3, EP12‐ed) for assay precision and reproducibility.
2. *Longitudinal variability*: Longitudinal variability was assessed in four cohorts including a surgical cohort (R0 and R1 patients: *n* = 25), patients with stable disease in a surveillance/watch‐and‐wait protocol (*n* = 20), patients currently being treated who have stable disease (*n* = 20 [1x everolimus; 2x PRRT; 17x SSA]) and 25 individuals with progressive disease. Samples were collected per clinical follow‐up (12–24 months). Standard imaging protocols (RECIST 1.1) were used to evaluate disease stability or progression. Scores were compared to the baseline score to evaluate how these changed with time. 1‐way ANOVA was conducted to determine score stability.

### 2.7 Statistical analysis

The predictive performance of the NETest 2.0 algorithms was assessed by using the AUROC curves. AUROCs were compared using DeLong test.^23^ Ninety‐five percent Confidence Intervals (CIs) were produced using 2000 bootstrap samples. Sensitivity, positive predictive value (PPV), and F1‐score (a measure of accuracy reflecting the harmonic mean of PPV and sensitivity, where one represents perfect PPV and sensitivity) were determined. Dimensionality reduction was performed using the non‐linear Ivis algorithm.^24^ Model probabilities were evaluated using the reliability diagram and the calibration_curve function in the scikit‐learn library. Predicted probabilities were binned into 10 discrete intervals, and the mean predicted probability and the true frequency of the positive class were plotted for each interval. Data are provided as median (range) and [mean ± SD]. Mann–Whitney *U*‐test was used to compare scores in the different cohorts, ANOVA (Kruskal–Wallis test) was used to evaluate longitudinal data and the McNemar's test was used for comparing NETest 2.0 with NETest 1.0. Prism 9.4 for Windows (GraphPad Software Inc., La Jolla, CA; www.graphpad.com) and MedCalc Statistical Software v20.110 (MedCalc Software Ltd., Ostend, Belgium; http://www.medcalc.org; 2017). Statistical significance was defined as *p* < .05. Statistical significance was defined as *p* < .05.

## 3 RESULTS

### 3.1 Supervised classifier development—NETest 2.0 Algorithm #1. Differentiation of controls and NETs

#### 3.1.1 In‐sample model (test set) performance

Raw C_t_ values were independently normalized to four housekeeping genes (*ALG9*, *ATG4B*, *RHOA*, and *TXNIP*). For each normalized training set, four classifiers were fitted to 192 blood samples (62 Controls, 101 Stable Disease, 29 Progressive, Figure S2). The best combination of hyperparameters and model performance was evaluated using leave‐one‐out cross‐validation (Figure S3). The best‐performing classifier in the in‐sample validation was RF, normalized to *ATG4B* (AUROC 0.901 [95% CI 0.899–0.902]), with a maximum depth of 8 and number of trees at 500, due to the highest achieved AUROC. Details regarding performances of the individual house‐keeping genes and algorithms are included in Table S7.

#### 3.1.2 Held‐out model performance (test set)

**FIGURE 2:**
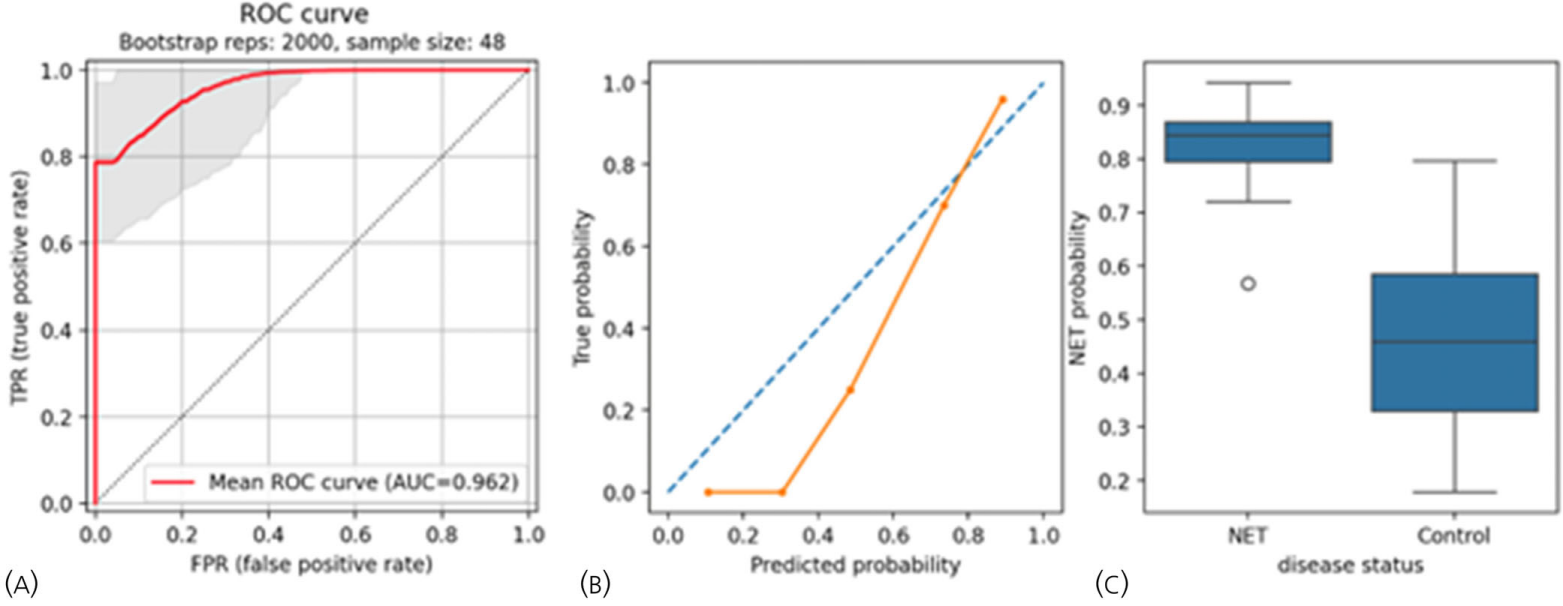
NETest 2.0 model performance on the out‐of‐sample testing dataset. (A) AUROC curve demonstrating discriminating performance between NETs and Controls. (B) Calibration curve comparing predicted versus actual NET probability. (C) Boxplots showing model probability values across NETs (*n* = 32) and Controls (*n* = 16). The boxes represent the quartiles of the dataset, while the whiskers extend to display the rest of the distribution. Outliers are shown as circles. NET, neuroendocrine tumors; ROC, receiver operating characteristic; AUC, area under the curve.

The RF classifier trained on *ATG4B*‐normalized gene expression was retained for a held‐out dataset evaluation (16 Controls, 25 Stable Disease, 7 Progressive Disease). The model achieved an AUROC of 0.962 (95% CI: 0.961–0.963, Figure 2A), was well‐calibrated (Pearson's *R* ^2^ = 0.965; *p* = .007) (Figure 2B) and the average model probabilities for NET were 0.82 (± 0.07) and Control groups 0.48 (± 0.19) respectively (Figure 2C).

Model performance was not affected by age or ethnicity (DeLong's *p*‐value = .74–.83, Figure S6A,B) but the AUROC was significantly smaller for patients in the first age quartile (33–51 years old, AUROC 0.87, DeLong's *p*‐value = .04, Figure S6C, Table S8).

### 3.2 Supervised classifier development—Algorithm #2. Differentiation of stable and progressive disease

#### 3.2.1 In‐sample model performance

Raw C_t_ values were independently normalized to four housekeeping genes (*ALG9*, *ATG4B*, *RHOA*, and *TXNIP*). For each normalized training set, four classifiers were fitted to 156 blood samples (107 Stable Disease, 49 Progressive, Figure S4). Hyperparameter selection for each classifier was performed using five‐fold cross‐validation, with in‐sample performance assessed using leave‐one‐out cross‐validation on the training set. The best combination of hyperparameters and model performance was evaluated using leave‐one‐out cross‐validation (Figure S5).

The best‐performing classifier in the in‐sample validation was RF, normalized to *ALG9* (AUROC 0.809 [95% CI 0.805–0.809]), with a maximum depth of 8 and number of trees at 1000, due to the highest achieved AUROC. Details regarding performances of the individual house‐keeping genes and algorithms are included in Table S9.

#### 3.2.2 Held‐out model performance (testing set)

**FIGURE 3:**
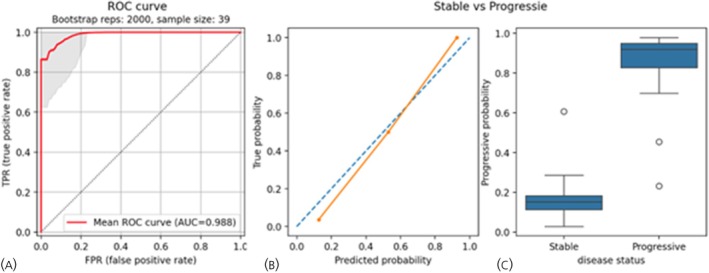
NETest 2.0 model performance on the out‐of‐sample testing dataset. (A) AUROC curve demonstrating the discriminating performance between Progressive and Stable disease samples. (B) Calibration curve comparing predicted versus actual Progressive probability. (C) Boxplots showing model probability values across Stable (*n* = 27) and Progressive NETs (*n* = 12). The boxes represent the quartiles of the dataset, while the whiskers extend to display the rest of the distribution. Outliers are shown as circles. NET, neuroendocrine tumors; ROC, receiver operating characteristic; AUC, area under the curve.

The RF classifier trained on *ALG9*‐normalized gene expression was retained for evaluation on a held‐out dataset (27 Stable Disease, 12 Progressive Disease). The model achieved an AUROC of 0.988 (95% CI: 0.987–0.988, Figure 3A) was well‐calibrated (Pearson's *R* ^2^ = 0.99; *p* = 0.02) (Figure 3B) and the average model probabilities for Progressive were 0.82 (±0.07) and Stable were 0.48 (±0.19), respectively (Figure 3C).

Model performance was not affected by gender, ethnicity, or age (DeLong's *p*‐value = 0.84–0.96, Figure S7A–C, Table S10).

### 3.3 NETest 2.0 model validation

Both algorithms were evaluated in independent testing sets, Cohort #I (277 NETs, 186 Controls), Cohort #II (291 NETs), Evaluation Cohort #I (147 other cancers), Evaluation Cohort #II (19 SCLC) and Evaluation Cohort #III (50 IPF) (Figure S1).

#### 3.3.1 Validation of NETest 2.0 Algorithm #1: Controls versus NETs

**FIGURE 4:**
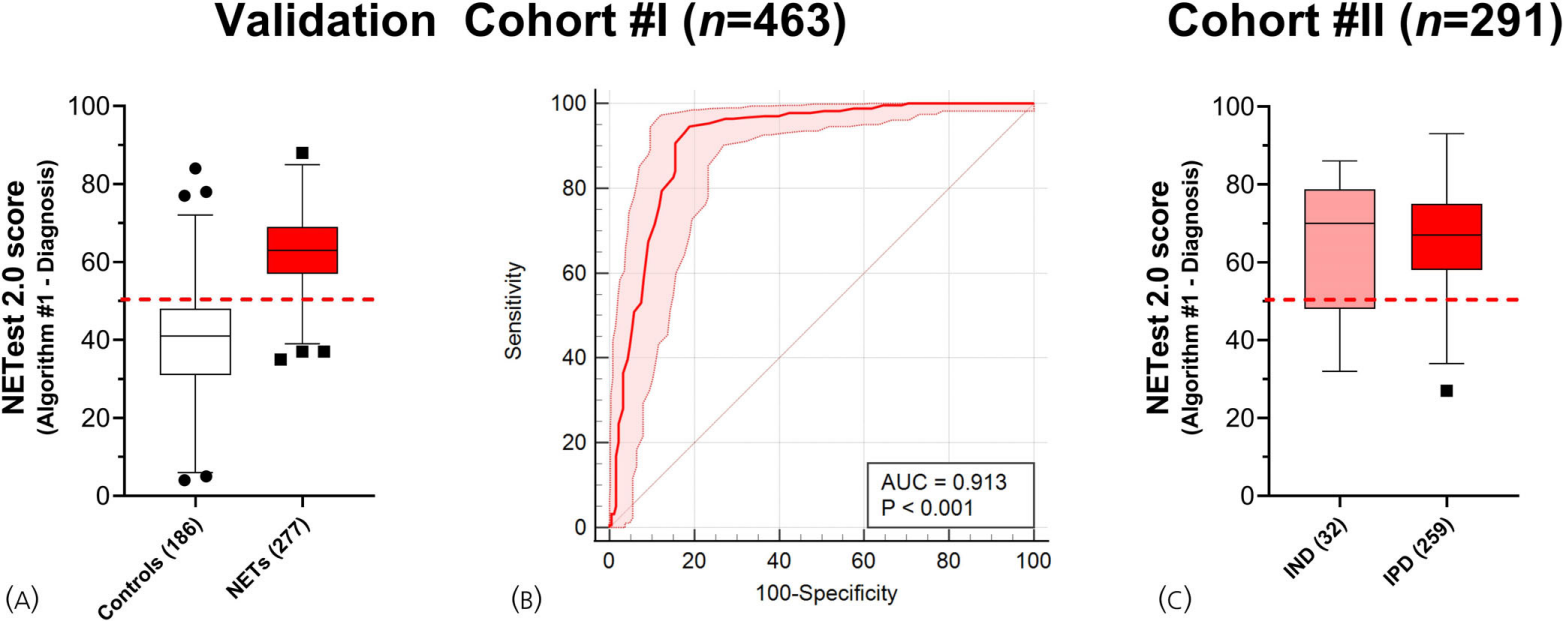
NETest 2.0 Algorithm #1 performance in two independent validation cohorts. (A). Box and whisker (Tukey) plot of NETest scores in Controls versus NETs (Cohort #I). Median and interquartile ranges are provided. (B) Receiver Operating Characteristic Curve for differentiating NETs from Controls in Cohort #I (*n* = 463). Shaded areas represent 95% CI. (C) Box and whisker (Tukey) plot of NETest scores in Cohort #II in the IND and IPD groups. Median and interquartile ranges are provided. Con, controls; NET, neuroendocrine tumors; AUC, area under the curve; IND, image‐non‐detectable; IPD, image‐positive‐disease. Red dashed line is the cut‐point for NET detection (50).

In Cohort #I, NETest scores for NETs was 63 ± 9, and for controls, it was 39 ± 16 (*p* < .0001, Mann–Whitney U‐test) (Figure 4A). NETs were differentiated from controls with an AUROC of 0.91 (95% CI: 0.87–0.94, Figure 4B). Setting the operating point to NET probability ≥50%, the NETest achieved a sensitivity of 94.6% (91%–97%), specificity of 81% (75%–87%), and PPV of 88% (85%–91%) (Table 2).

**TABLE 2:** Diagnostic metrics for the NETest (Algorithm #1) in validation Cohort #I (*n* = 463).

Statistic	Value	95% CI
Sensitivity	94.6%	91.2%–96.9%
Specificity	81.2%	74.8%–86.5%
PPV^a^	88.2%	84.7%–91.0%
NPV^a^	91.0%	86.0%–94.3%
Accuracy^a^	89.2%	86.0%–91.9%

^a^ These values are dependent on disease prevalence.

In Cohort #II, the NETest was positive in 260/291 (89.3%) of individuals (Figure 4C). The mean score was 66 ± 12. Two hundred and thirty‐eight (92%) of 259 NETs with image‐detectable/positive disease (IPD) were NETest‐positive. Twenty‐two (69%) of 32 NETs with no image‐detectable disease were also NETest‐positive.

#### 3.3.2 Specificity Evaluation of NETest 2.0 Algorithm #1: Other malignancies and non‐malignancy diagnoses

**FIGURE 5:**
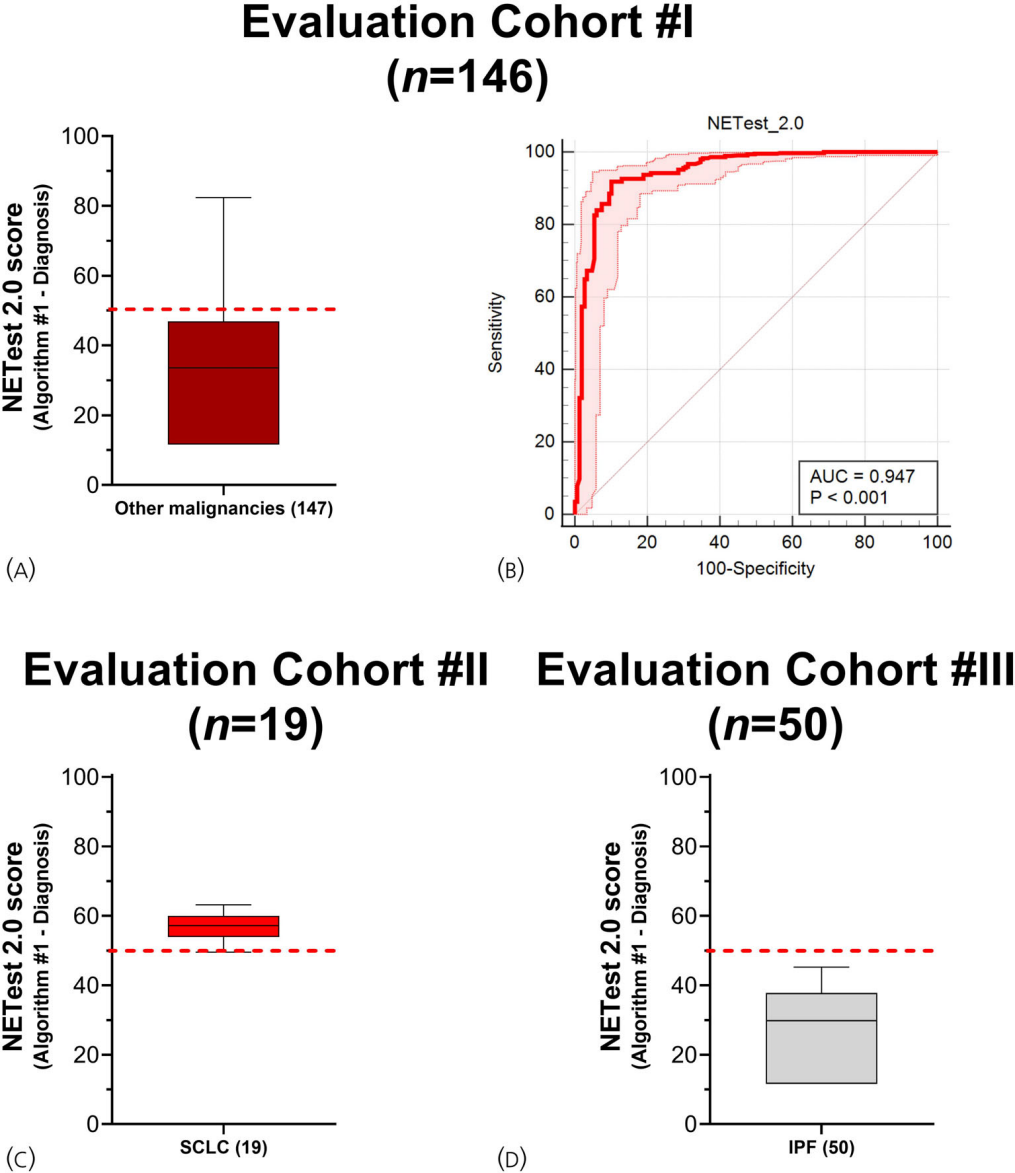
NETest 2.0 Algorithm #1 performance in other diseases. (A) Box and whisker (Tukey) plot of NETest scores in other cancers (Evaluation Cohort #I). (B) Receiver Operating Characteristic Curve for differentiating NETs (Cohort I + II, *n* = 568) from other cancers (*n* = 147, Evaluation Cohort #I). (C) Box and whisker (Tukey) plot of NETest scores in Evaluation Cohort #II (SCLC). (D) Box and whisker (Tukey) plot of NETest scores in Evaluation Cohort #III (IPF). AUC, area under the curve; IPF, interstitial pulmonary fibrosis; SCLC, small cell lung cancer. Median and interquartile ranges are provided. Red dashed line is the cut‐point for NET detection (50). Shaded areas represent 95% CI for the AUROC plot.

The NETest was positive in 15/147 (10.2%) of individuals with a non‐NET malignancy (Evaluation Cohort #I, Figure 5A). NETs were differentiated from these malignancies with an AUROC of 0.95 (95% CI: 0.93–0.96, Figure 5B). The sensitivity was 91.9% (89.4%–94%), specificity of 90% (83.7%–94.2%), and PPV of 97% (95.6%–98.3%) at the operating point ≥50% (Table 3). In other GI and pancreatic malignancies (*n* = 85), the NETest was positive in 7 (8.3%). For lung malignancies (*n* = 59), the NETest was positive in 5 (8.5%, Table S11).

**TABLE 3:** Diagnostic metrics for the NETest (Algorithm #1) for differentiating NETs from other malignancies (Cohort #III).

	Validation Cohort #III (*n* = 147)	
Statistic	Value	95% CI
Sensitivity	91.90%	89.35%–94.01%
Specificity	89.80%	83.73%–94.18%
PPV^a^	97.21%	95.56%–98.25%
NPV^a^	74.16%	68.39%–79.19%

^a^ These values are dependent on disease prevalence.

The NETest was positive in 18/19 (94.7%) of individuals with a SCLC diagnosis (Evaluation Cohort #II; Figure 5C) and was negative in all individuals with IPF (Evaluation Cohort #III, Figure 5D).

#### 3.3.3 Validation of NETest 2.0 Algorithm #2: Stable versus progressive disease

**FIGURE 6:**
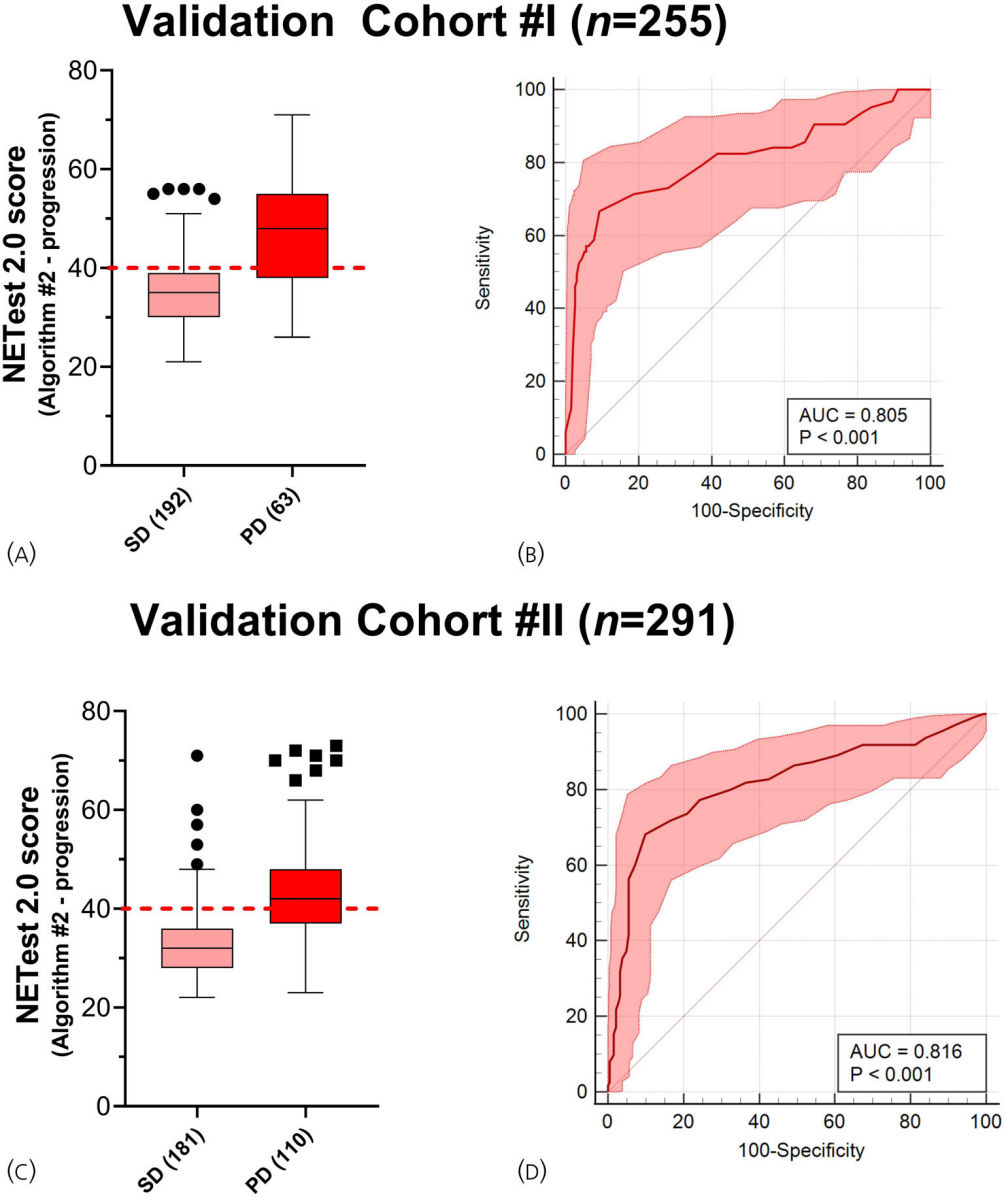
NETest 2.0 Algorithm #2 performance in two independent validation cohorts. (A) Box and whisker (Tukey) plot of Scores in Progressive versus Stable disease in the Validation Cohort #I. Median and interquartile ranges are provided. (B) ROC Curve for differentiating Progressive from Stable disease in Validation Cohort #I. Shaded areas represent 95% CI. (C) Box and whisker (Tukey) plot of Scores in Progressive versus Stable disease in Validation Cohort #II. Median and interquartile ranges are provided. (D) ROC Curve for differentiating Progressive from Stable disease in Validation Cohort #II. Shaded areas represent 95% CI. SD, stable disease; PD, progressive disease; AUC, area under the curve. Red dashed line is the cut‐point for progressive disease (40).

In the Validation Cohort #I, using the same blood sample, progression scores for Progressive disease (*n* = 63) was 46 ± 11, and for Stable disease (*n* = 192), it was 35 ± 7 (*p* < .0001, Mann–Whitney U‐test) (Figure 6A). Progressive disease was differentiated from Stable disease with an AUROC of 0.81 (95% CI: 0.75–0.85, Figure 6B). Setting the operating point to Progressive probability ≥40%, the NETest 2.0 Algorithm #2 achieved a sensitivity of 71% (59%–82%), specificity of 81% (75%–86%), and PPV of 56% (47%–64%) for differentiating Progressive from Stable disease (Table 4).

**TABLE 4:** Diagnostic metrics for the NETest (Algorithm #2) in each of the two validation cohorts.

	Validation Cohort #I (*n* = 255)		Validation Cohort #II (*n* = 291)	
Statistic	Value	95% CI	Value	95% CI
Sensitivity	71.4%	58.7%–82.1%	63.9%	53.54%–73.4%
Specificity	81.3%	75.0%–86.5%	87.8%	81.1%–92.7%
PPV^a^	55.6%	47.3%–63.6%	78.5%	69.5%–85.4%
NPV^a^	89.7%	85.4%–92.8%	77.7%	72.6%–82.1%

^a^ These values are dependent on disease prevalence.

In Validation Cohort #II, Progression scores for Progressive disease (*n* = 110) was 43 ± 11, and for Stable disease (*n* = 181), it was 33 ± 7 (*p* < .0001, Mann–Whitney U‐test) (Figure 6C). The AUROC was 0.82 (95% CI: 0.77–0.84, Figure 6D). Setting the operating point to ≥40%, a sensitivity of 81% (71%–88%), a specificity of 82% (76%–87%), and PPV of 68% (61%–55%) was achieved for differentiating Progressive from Stable disease (Table 4).

### 3.4 Model evaluation

The impacts of different cut‐points (e.g., 10%, 20%⋯50%⋯89%, 90%) were examined for each of the two algorithms. For these analyses, Cohort I and II were combined.

1. *NETest 2.0 Algorithm #1—Diagnostic algorithm* (*Controls: n* = *186; NETs: n* = *568*): Line plots of model prediction behavior for Algorithm #1 are included in Figure S8A. Individual metrics for each cut‐point are included in Table S12. An operating point of 50% provided the best metrics for differentiating NETs (50%–100%) from controls (0%–49%).
2. *NETest 2.0 Algorithm #2—Prognostic algorithm* (*SD: n* = *373; PD: n* = *173*): Line plots of model prediction behavior for Algorithm #2 are included in Figure S8B. Individual metrics are included in Table S13. An operating point of 40% was identified as providing the best metrics for differentiating Progressive from Stable disease.

The impact of gender, ethnicity and age on model performance was evaluated for both algorithms. In a subgroup analyses, model performance was not affected by gender, ethnicity, or age. For Algorithm #1 (*n* = 754), this is included in Table S14; for Algorithm #2 (*n* = 546), see Table S15.

### 3.5 Assay metrics—“Real world” evaluation

*Intra‐ and inter‐assay variability*: The individual reproducibility metrics are included in (Table 5). The overall intra‐assay variability for the 78 paired samples (78 PCR runs) was 0.55% and the inter‐assay variability in 146 PCR runs was 0.61%. No statistically significant differences were noted in intra‐ and inter‐assay variabilities between individual operators (*p* = 0.53–0.57), machines (*p* = 0.24–0.94), lots (*p* = 0.32–0.74) or on different days (*p* = 0.09–0.97).

**TABLE 5:** Intra‐ and inter‐assay metrics.

	Intra‐assay variability		Inter‐assay variability	
Overall	0.55% ± 0.065%		0.68% ± 0.088%	
Operator 1 versus Operator 2	0.25%	0.77%	1.9%	1.81%
Machine 1 versus Machine 2	0.52%	0.58%	1.77%	1.89%
Lot 1 versus Lot 2^a^	0.38%	0.41%	1.88%	1.75%
Day‐to‐day variation^b^	KW‐statistic: 2.8, *p* = .97		KW‐statistic: 13.5, *p* = .10	

*Note*: These are included for each of the studies (different operators, machines and lots as well as day‐today variability).

Abbreviation: KW, Kruskal–Wallis statistic.

^a^ NETest spotted plate lots: #8294149; 8325911.

^b^ Run over a 20‐day period.

**FIGURE 7:**
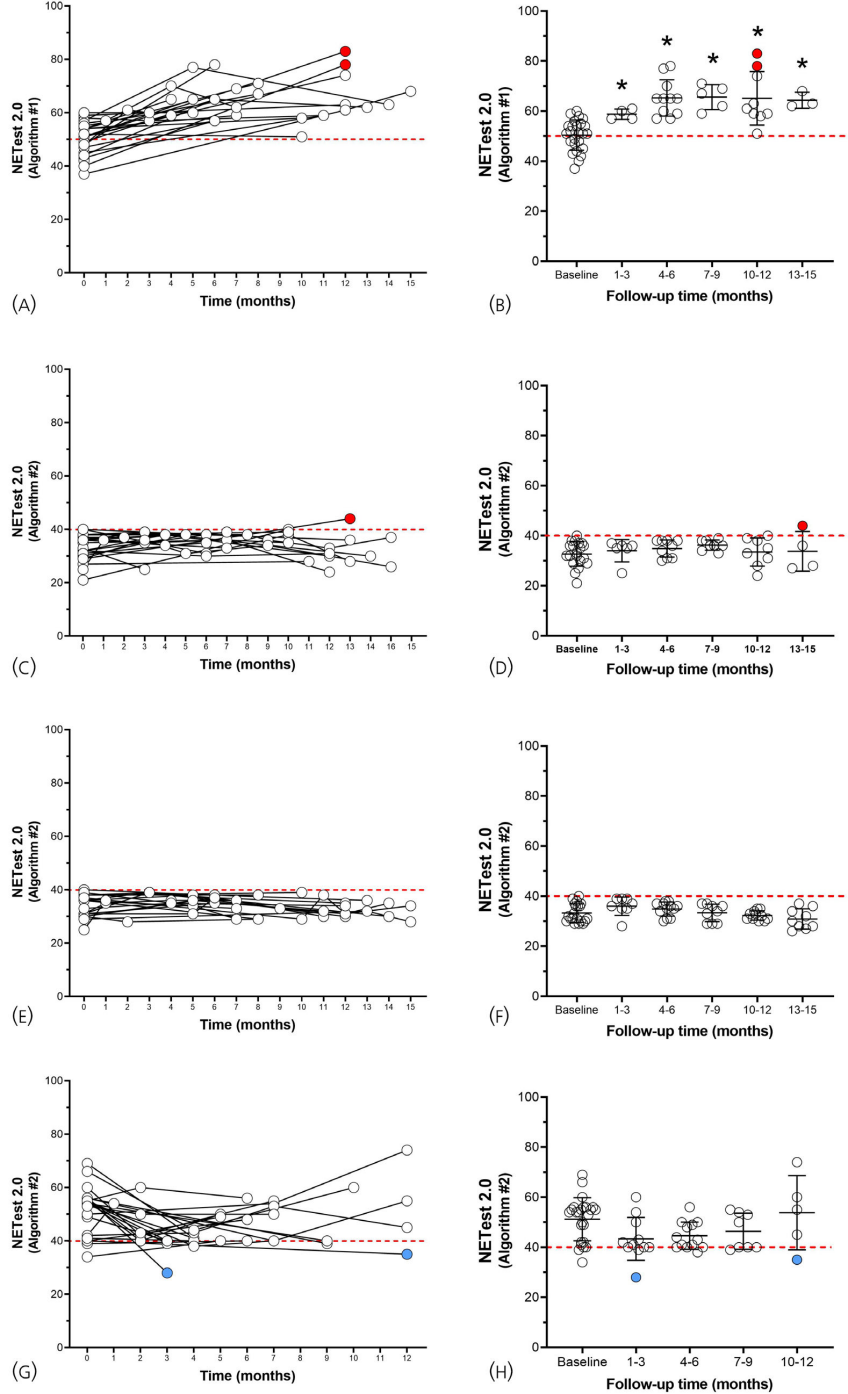
Longitudinal evaluation of NETest scores (Algorithm #1 and Algorithm #2) in 4 different cohorts. (A, B) Follow‐up in 25 surgically resected patients with either no positive margins (R0) or microscopic residual disease (R1). Scores (Algorithm #1—Diagnostic) increase with time since surgery. Two patients (red circles) developed image‐detectable disease during the follow‐up period. (C, D) In 20 patients with clinically stable disease [watch & wait/surveillance], the prognostic score (Algorithm #2) was stable (<40) over the follow‐up. One patient's score (red circle) was >40 concordant with the development of disease progression. This was confirmed on imaging. (E, F) In 20 patients undergoing treatment, Algorithm #2 scores were all <40 consistent with disease stability (and response to therapy). (G, H) In 25 patients with progressive disease, scores were >40 in 90% of cases. Two patients responded to therapy with disease stabilization (blue circles). Both exhibited scores <40 consistent with good therapeutic responses. Individual scores (A, C, E, and G) and scores grouped in 3 monthly intervals (B, D, F, and H) are included. (A, B) Red dashed line is the cut‐point for NET detection (50). (C–H) Red dashed line is the cut‐point for progressive disease (40).

*Longitudinal variability*: Variability in Algorithm #1 was assessed a surgically treated cohort (*n* = 25), while variability in Algorithm #2 was assessed in individuals with stable disease (*n* = 20) in a watch & wait/surveillance program, individuals with stable disease (*n* = 20) who are currently being treated, and those with progressive disease (*n* = 25). Algorithm #1 demonstrated an increase in scores from baseline (Kruskal–Wallis statistic: 38.8, *p* < .0001) with elevation in scores between 4 and 15 months after surgery (Figure 7A,B). This is consistent with identification of residual disease in these patients. Two of the patients with positive scores (>70), developed image detectable disease at 12 months follow‐up. Algorithm #2 did not exhibit significant variability in either of the two “stable” disease cohorts (KW statistic: 3.7, *p* = 0.59 and 1.2, *p* = 0.48, respectively, Figure 7C–F). One patient in the untreated cohort developed progressive disease. This patient exhibited a progressive score of 44 at time of evaluation. In individuals with progressive disease, scores were significantly different over the 12‐month time‐frame (KW statistic: 9.51, *p* = 0.05, Figure 7G,H). This is consistent with therapeutic intervention and responses.

### 3.6 Algorithm comparison

As a final step, we directly compared the diagnostic metrics of NETest 2.0 with NETest 1.0. This was undertaken in the combined Validation Cohort #I and #II.

#### 3.6.1 NETest 2.0 Algorithm #1 versus NETest 1.0 (*n* = 754)

Model performance of each of the two algorithms is shown in Table 6.

**TABLE 6:** Comparison of diagnostic metrics for the NETest 2.0 (Algorithm #1 and Algorithm #2) versus the original NETest output in the combined validation cohorts.

	NETest 1.0		NETest 2.0	
Statistic	Diagnostic	Prognostic	Diagnostic	Prognostic
Sensitivity	79% (76%–82%)	32% (25%–40%)	92% (89%–94%)	69% (62%–76%)
Specificity	52% (44%–59%)	69% (64%–73%)	81% (75%–87%)	86% (82%–89%)
PPV^a^	83% (81%–85%)	33% (28%–39%)	94% (92–95%)	69% (63%–74%)
NPV^a^	47% (39%–50%)	68% (65%–71%)	77% (71%–81%)	86% (83%–88%)
Accuracy^a^	72% (69%–76%)	57% (53%–61%)	89% (87%–91%)	80% (77%–84%)

^a^ These values are dependent on disease prevalence.

For the diagnostic performance, NETest 2.0 (cut‐off 50) was superior to NETest 1.0 (cut‐off 20), regarding all metrics tested. The updated diagnostic algorithm significantly outperformed NETest 1.0 (McNemar's test, *χ* ^2^ = 36.2, *p* = 1.73 × 10^−9^).

#### 3.6.2 NETest 2.0 Algorithm #2 versus NETest 1.0

In terms of prognostic performance, NETest 2.0 (cut‐off 40) also showed substantial improvement over NETest 1.0 (cut‐off 40). All metrics were superior for the updated algorithm and NETest 2.0 significantly outperformed NETest 1.0 (McNemar's test, *χ* ^2^ = 41.8, *p* = 1.02 × 10^−10^).

## 4 DISCUSSION

This paper details the development and validation of two new classifiers for an established, clinically validated molecular assay used in NET disease management, the NETest. The updated classifiers, NETest 2.0 (Algorithm #1 and Algorithm #2) demonstrate improved discrimination and calibration performance in differentiating NETs from controls and distinguishing stable from progressive disease compared to the original algorithm. Specifically, the new algorithms are more accurate for detecting a NET, can differentiate NETs from non‐NET malignancies (and have fewer false positives), provides a more stable longitudinal readout and functions more accurately for detecting the disease status.

The original NETest algorithm provided reliable diagnostic metrics for patients with neuroendocrine tumor disease^7^, ^9^, ^25^ but several studies identified “false” positives.^8^, ^9^, ^12^, ^13^ In one study,^8^ 7 of 21 (33%) GI malignancies were positive. Most 5/7 [71.4%] were adenocarcinomas of the colon. In a second study 17/37 (46%) of colon cancers were NETest‐positive.^13^ In the current study, using an updated algorithm, 7/85 (8.2%) of GI malignancies were NETest‐positive. In the colon cohort, 3/47 (6.4%) were positive. This identifies a significant improvement in diagnostic differentiation (24/58 vs. 7/85: *χ* ^2^ test: 22.1, *p* < 0.0001) for NETest 2.0 and non‐NET GI malignancies. Other studies have evaluated the NETest in lung malignancies and other diseases, for example, IPF.^12^, ^13^ In one study 17/41 (42%) with lung adenocarcinomas and 13/37 (35%) of lung squamous cell cancers were NETest‐positive using the original algorithm. In the current study, NETest 2.0 was positive in 4/53 (7.5%) adenocarcinomas and 1/6 SCC. This demonstrates the updated algorithm is also effective for differentiating lung NETs from other non‐NET malignancies (30/78 vs. 5/59: *χ* ^2^ test: 15.8, *p* = 0.0001). Subjects with IPF have been identified to exhibit NETest‐positive in 18/50 (36%) of cases.^13^ All were negative using the updated NETest 2.0 algorithm. We also examined SCLC, which are classified as a high‐grade neuroendocrine cancer. These tumors are considered to have a neuroendocrine genotype.^26^ Eighteen of 19 (94.7%) were positive consistent with the NETest 2.0 effectively detecting this type of neuroendocrine tumor.

The NETest 2.0 is significantly more accurate than the original NETest algorithm and has significantly fewer false positives. While this identifies it is effective for detecting NETs, the metrics (PPV: 94%; NPV: 77%) and potential costs of deploying this molecular assay identify it should not be considered for use as a population‐based screening tool. We consider three possibilities for potential clinical utility. The first is in detection of minimal residual disease after “curative” surgery. Between 12% and 40% of lung and GEP‐NETs recur after a R0 surgery.^27^, ^28^ Guidelines have been developed to follow‐up patients (imaging, symptoms) but are not commonly followed.^29^ A positive NETest is known to detect microscopic disease.^30^, ^31^ We hypothesize that the diagnostic algorithm may have similar utility. Indeed, in a small cohort of 20 patients with microscopic disease, 2 (10%) developed image‐positive disease and were NETest positive. A previous evaluation of NETest 1.0 in a Dutch cohort (*n* = 30, all no image evidence of disease), identified that consecutive NETest scores (collected over a 54‐month period) fluctuated substantially (range: 0–100).^14^ The variability in NETest 2.0 diagnostic scores in our study of 25 R0/R1 patients was low (7.3%). Utility will require formal prospective evaluation with linkage to standardized, guideline‐approved imaging protocols for this group of NETs. The second area is in stratification for follow‐up in high‐risk patients. Subjects with clinical symptoms (often non‐specific) or a CT/MRI/FDG‐PET positive lesion could be considered as candidates for a NETest. It is possible that a positive NETest could be used as a stratification tool for further imaging studies (e.g., ^68^Ga/PET‐SSA imaging) or for biopsy/surgery for a definitive tissue‐based diagnosis. This requires formal investigation. The third area of potential utility would be in patients with known germline mutations, for example, MEN‐1. A positive test could possibly indicate the presence of a lesion, ensuring that these high‐risk patients are identified early for more detailed diagnostic workups. This potential utility would also require evaluation in a formal, longitudinal prospective study.

The original NETest algorithm had utility for differentiating stable from progressive disease.^9^, ^32^, ^33^, ^34^ Higher scores are typically identified in individuals with disease progression, and this has prognostic (progression free‐ and overall survival) relevance.^32^, ^33^ Omic indices (genes related to, e.g., proliferation),^11^, ^22^ were used in the initial algorithm to identify progressive disease. In NETest 2.0, we specifically utilized gene expression (including omes) to build a “diagnostic” tool for disease progression. This was defined as individuals exhibiting clinical and/or image‐based evidence of progression. In head‐to‐head comparisons, this algorithm (NETest 2.0 Algorithm #2) was identified to be more accurate (80% vs. 60%) than the original algorithm. The sensitivity and specificity have been increased through the development of an algorithm specifically trained to detect disease status. We anticipate that the updated version will provide added utility to the assay as a tool in this regard.

One use of the NETest is in monitoring patients who are either undergoing a treatment or are in a “surveillance” program. The original NETest algorithm exhibited value in this regard but consecutive scores have fluctuated over time in individuals with stable disease or no evidence of disease.^14^ We present preliminary information from 3 cohorts, a watch‐and‐wait, a stable disease cohort undergoing treatment (predominantly SSAs) and a progressive cohort. These 65 patients were followed for ~15 months and their clinical status evaluated per standard protocols. Individuals who developed progressive disease (either during surveillance or on treatment) all were identified with elevated prognostic scores during this period. Conversely, those who remained stable exhibited scores below 40. Consecutive measurements where very stable irrespective of the timing of the blood collections and no fluctuations were identified (the variability was low: 10.2%–15%). Patients who were progressive and did respond to therapy all exhibited scores <40 consistent with disease “stabilization.” These data indicate a potential role for the NETest in monitoring patients in different settings but this requires a prospective clinical trial for validation.

This study has several strengths: it was built on the same platform as PROSTest,^5^ ensuring methodological consistency, and it utilized significantly more clinical samples than were included in the development and validation of NETest 1.0. The patient and control sets were expanded for more comprehensive development and validation, including samples from subjects previously determined as false positive (e.g., GI malignancies, lung adenocarcinomas, IPF), and the study included a direct comparison with state‐of‐the‐art imaging (^68^Ga‐/^64^Cu‐somatostatin receptor PET), highlighting its diagnostic accuracy. The algorithms were validated using globally collected samples, suggesting their broader applicability, and the results were not affected by patient age, ensuring reliability across different age groups. However, there are some limitations: ethnicity evaluations may need further expansion, requiring more diverse patient numbers. Nevertheless, the test/algorithms work effectively in all ethnic groups. This study was undertaken in subjects world‐wide so includes a mix of well‐characterized and less‐well characterized individuals. The value of the test in tumors from different locations, at different TNM stages and in different grades cannot be undertaken. A separate examination of such a well‐characterized cohort is required to evaluate test metrics in different conditions. Finally, this manuscript provides evidence for assay development and provides metrics for reproducibility per guidelines. Formal prospective studies are required to validate clinical utility of the two algorithms.

**FIGURE 8:**
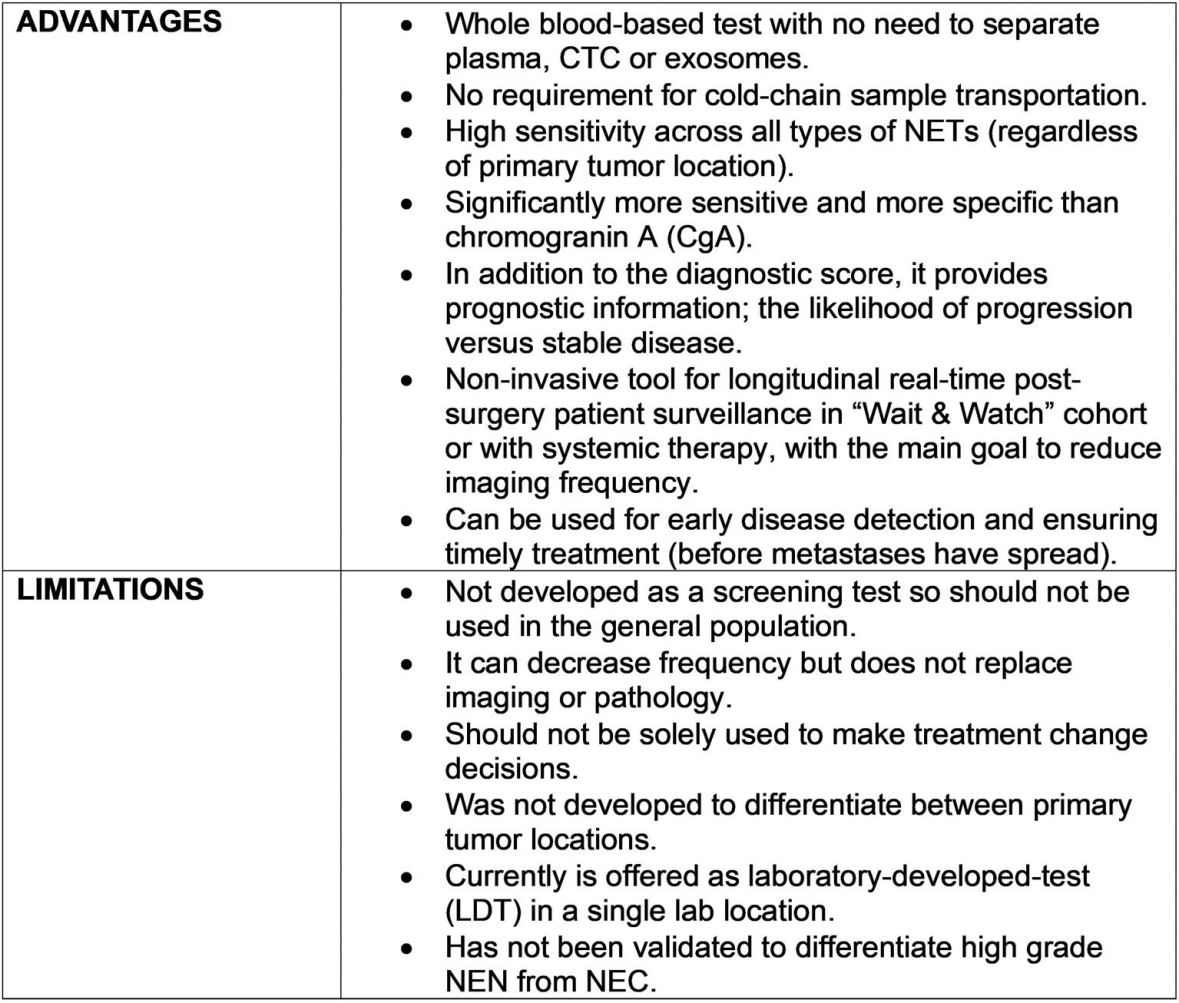
Advantages and limitations of the NETest.

In conclusion, the NETest 2.0 algorithms provides a significant advancement compared to the original algorithm (Table 1). NETest 2.0 was built and validated using RNA‐stabilized blood samples, which were collected world‐wide. The algorithms and mathematical approaches have been updated and the outputs (scores) have been simplified for easier interpretation and clinical application. In terms of practical guidance for use in the clinic, we consider the test does not replace pathology and imaging but provides additional information to the clinician and patient. The test offers the following advantages and limitations (Figure 8). The advantages range from meeting an unmet need for an accurate, reliable biomarker for disease surveillance to the possibility of reducing the amount of imaging a patient undergoes while the limitations range from the fact that the tool was not developed for general screening (and should not be used for population evaluation) to the fact that there is currently limited information for its value in high grade NEN and NECs. Overall, compared to the well‐studied NETest 1.0, NETest 2.0 exhibits enhanced sensitivity and specificity. The high precision (intra‐/inter‐assay variabilities) and reproducible scores during follow‐up, identify a more reliable tool for NET management.

## AUTHOR CONTRIBUTIONS

**M. Kidd:** Conceptualization; investigation; writing – original draft; methodology; validation; visualization; writing – review and editing; project administration; data curation. **I. A. Drozdov:** Conceptualization; investigation; writing – original draft; methodology; validation; visualization; writing – review and editing; software; formal analysis; data curation. **A. Chirindel:** Investigation; funding acquisition; writing – original draft; writing – review and editing; methodology; project administration; data curation; resources; formal analysis. **G. Nicolas:** Data curation; resources; project administration; formal analysis; writing – review and editing; methodology; investigation; funding acquisition; writing – original draft. **D. Imagawa:** Investigation; writing – original draft; validation; writing – review and editing; formal analysis; project administration; resources; data curation. **A. Gulati:** Investigation; writing – original draft; validation; visualization; writing – review and editing; formal analysis; project administration; resources; data curation. **T. Tsuchikawa:** Investigation; funding acquisition; writing – original draft; methodology; validation; writing – review and editing; project administration; data curation; resources. **V. Prasad:** Writing – original draft; methodology; validation; visualization; writing – review and editing; investigation. **A. B. Halim:** Conceptualization; investigation; funding acquisition; writing – original draft; methodology; validation; visualization; writing – review and editing; formal analysis; project administration; supervision; resources. **J. Strosberg:** Investigation; writing – original draft; methodology; validation; writing – review and editing.

## CONFLICT OF INTEREST STATEMENT

MK and AH are employees of Wren Laboratories. IAD has consulted for Wren Laboratories and is a shareholder at Bering Limited. JS has received research support from ITM, Novartis, Radi‐omedix and RayzeBio and has consulted for Boehringer‐Ingelheim. AC, GC, DA, AG, TT and VP have no conflicts.

### PEER REVIEW

The peer review history for this article is available at https://www.webofscience.com/api/gateway/wos/peer‐review/10.1111/jne.70002.

## Supporting information


Data S1.

## Data Availability

The data that support the findings of this study are available on request from the corresponding author. The data are not publicly available due to privacy or ethical restrictions.
