# Supplementary material for: NETest® 2.0—A decade of innovation in neuroendocrine tumor diagnostics
Source: J Neuroendocrinol. 2025 Feb 13;37(4):e70002. doi: 10.1111/jne.70002 (PMC11975799; doi:10.1111/jne.70002)
Supplement: Supplementary file 1 — Data S1. [file JNE-37-e70002-s001.docx]

**NETest® 2.0 – A Decade of Innovation in**

**Neuroendocrine Tumor Diagnostics**

Kidd M^1^*, Drozdov IA^2^, Chirindel A^3^, Nicolas G^3^, Imagawa D^4^, Gulati A^5^, Tsuchikawa T^6^,

Prasad V^7^, Halim AB^1*^, Strosberg J^8^

**Affiliations:**

^1^Wren Laboratories, Branford CT

^2^Bering Research, London UK

^3^University of Basel, Basel Switzerland

^4^University of California – Irvine, Orange, CA

^5^Bennett Cancer Center, Stamford, CT

^6^Hokkaido University Hospital, Sapporo, Japan

^7^Mallinckrodt Institute of Radiology, School of Medicine, Washington University in St. Louis, St. Louis, MO

^8^Moffitt Cancer Center, Tampa FL

**Running title:** NETest 2.0

***Corresponding Authors:**

Abdel Halim, PharmD, PhD Mark Kidd PhD

[ahalim@wrenlaboratories.com](mailto:ahalim@wrenlaboratories.com) [mkidd@wrenlaboratories.com](mailto:mkidd@wrenlaboratories.com)

**Supplementary Information**

**Gene Expression Measurements in Whole Blood**

An overview of the populations, sites and times of collection for assay development and validations are included in **Supplementary Figure S1**.

**
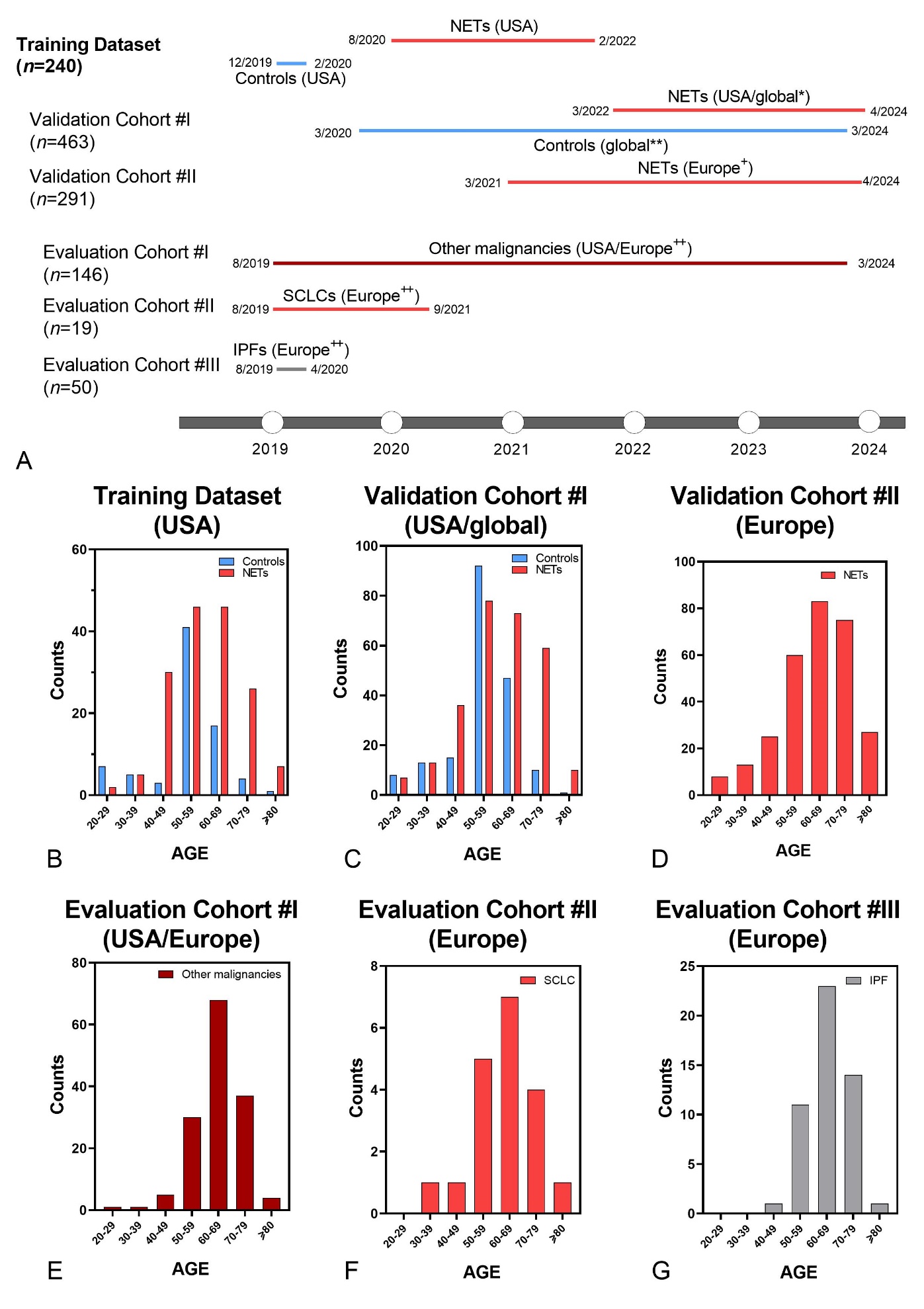
Supplementary Figure S1. Overview of populations used for the assay development**

**S1A)** Timeline of sample collection. Each horizontal line represents the collection period of samples assigned to training and validation datasets**. S1B-G)** Histograms of age distributions across all datasets.

*global includes Latin America and Asia

**global includes Asia, Africa, Europe, Latin America and the USA.

+Switzerland

++Poland

**Assay Development**

**Model (NETest) Training**

*Algorithm Development Dataset:* Gene expression was evaluated in blood samples from 240 subjects including 78 healthy volunteers and 162 NETs (**Supplementary Table S1**).

**Supplementary Table S1.** Demographics of Algorithm Development (Training – USA) Dataset (*n*=240) – Algorithm #1

|  | **Controls (*n*=78)** | **NET cohort**  **(*n*=162)** | | | ***p*-value** |
| --- | --- | --- | --- | --- | --- |
| **Age**  **Median (range)** | 55 (22-80) | 59 (24-92) | | | 0.017* |
| **Gender (M:F)** | 47:31 | 75:87 | | | 0.054^†^ |
| **Ethnicity**  **A:B:H:W** | 3:16:16:43 | 6:45:7:104 | | | 0.001^‡^ |
|  |  |  | | |  |
| **Location** |  | Lung:  Stomach:  Pancreas:  Small Bowel:  Appendix:  Colorectum:  CUP/other: | 24  8  44  61  3  12  10 | 14.8%  4.9%  27.2%  37.7%  1.9%  7.3%  6.2% | N/A |
| **Grade** |  | G1:  G2:  G3:  ND: | 73  68  9  12 | 45.1%  42.0%  5.6%  7.3% | N/A |
| **Stage** |  | Localized:  Metastatic:  ND: | 55  107  0 | 34%  66%  0% | N/A |
| **Clinical Status** |  | Stable:  Progressive: | 126  36 | 77.8%  22.2% | N/A |

*Mann-Whitney U-test (2-tailed); ^†^Fisher’s exact test; ^‡^Chi^2^ test.

A=Asian, B=Black, H=Hispanic, W=White.

CUP = carcinoid of unknown primary; ND = no data

*83 (51.2%) were in monitoring/surveillance, 68 (42%) were on an SSA, 5 (3.1%) were being treated with chemotherapy (CAPTEM), 2 (1.2%) were on an SSA+Everolimus, 2 (1.2%) were on everolimus and 2 (1.2%) on Sutent.*

*No patient was treatment-naïve.*

**Supplementary Table S2.** Demographics of Algorithm Development (Training – USA) Dataset (*n*=195) – Algorithm #2

|  | **Stable (*n*=134)** | | | **Progressive (*n*=61)** | | | ***p*-value** |
| --- | --- | --- | --- | --- | --- | --- | --- |
| **Age** | 60 (24-92) | | | 61 (25-87) | | | 0.73* |
| **Gender** | 57:77 | | | 36:25 | | | 0.044^†^ |
| **Ethnicity** | 5:29:6:94 | | | 1:16:1:43 | | | 0.586^‡^ |
|  |  | | |  | | |  |
| **Location** | Lung:  Stomach:  Pancreas:  Small Bowel:  Appendix:  Colorectum:  CUP/other: | 18  7  34  53  4  10  8 | 13.4%  5.2%  25.4%  39.6%  3%  7.4%  6% | Lung:  Stomach:  Pancreas:  Small Bowel:  Appendix:  Colorectum:  CUP/other: | 8  2  20  21  1  2  7 | 13.1%  3.3%  32.8%  34.4%  1.6%  3.3%  11.5% | 0.595^‡^ |
| **Grade** | G1:  G2:  G3:  ND: | 60  55  8  11 | 44.8%  41.0%  6.0%  8.2% | G1:  G2:  G3:  ND: | 30  24  6  1 | 49.2%  39.4%  9.8%  1.6% | 0.260^‡^ |
| **Stage** | Localized:  Metastatic: | 51  83 | 38.1%  61.9% | Localized:  Metastatic: | 11  50 | 18.0%  72.0% | 0.005^†^ |

*Mann-Whitney U-test (2-tailed); ^†^Fisher’s exact test; ^‡^Chi^2^ test.

A=Asian, B=Black, H=Hispanic, W=White.

CUP = carcinoid of unknown primary; ND = no data

*In those with stable disease: 68 (50.7%) were in monitoring/surveillance, 57 (42.5%) were on an SSA, 5 (3.7%) were being treated with chemotherapy (CAPTEM), 2 (1.5%) were on everolimus and 2 (1.5%) on Sutent.*

*In those with progressive disease: 38 (62.3%) were on an SSA, 19 (31.1%) were in monitoring/surveillance, 2 (3.3%) were being treated with an SSA+everolimus, 1 (1.6%) was on short-acting octreotide and no information was available in 1 (1.6%).*

*No patient was treatment-naïve.*

**NETest signature derivation: Omic information**

For feature engineering, individual “omes” were included in the algorithm (**Supplementary Table S3**). These are summated normalized gene expression of specific genes associated with specific biological functions. NETest omes were initially detailed in 2015 (1) and updated in 2021 (2).

**Supplementary Table S3**. Omes

- Apoptome: *BNIP3L, WDFY3*
- Epigenome: *MORF4L2, NAP1L1, PQBP1, RNF41, RSF1, SMARCD3, ZFHX3*
- Fibrosome (3): *APLP2, BNIP3L, CD59*
- Growth Factor Signalome: *ARAF, KRAS, BRAF, RAF1*
- Inflammasome: *CD59, PHF21A, PQBP1, SMARCD3*
- Metabolome: *ATP6V1H, OAZ2, PANK2, PLD3*
- Metastasome: *APLP2, FLJ1035, ATP6V1H, CD59, COMMD9, CTGF, NUDT3*
- NEDome: *AKAP8L, FLJ1035, FZD7, GLT8D1, HSF2, NAP1L1, OAZ2, PHF21A, PLD3, PQBP1, RNF41, SMARCD3, SPATA7, SSTR1, SSTR3, SSTR4, SSTR5, TPH1, VMAT1, ZFHX3, ZXDC*
- Neurome: *AKAP8L, APLP2, ENPP4, GLT8D1, HSF2, PANK2, PHF21A, PLD3, PQBP1, RTN2, TECPR2, TPH1, VMAT1, VMAT2, VPS13C, WDFY3, ZFHX3*
- Plurome: *COMMD9*
- Proliferome: *NAP1L1, NOL3, MKI67, TECPR2*
- Secretome (General): *PNMA2, VMAT2*
- Secretome (Progressive): *PQBP1, VMAT2*
- SSTRome: *SSTR1, SSTR3, SSTR4, SSTR5*
- Tfome: *ZFHX3, ZXDC, ZZZ3*
- ROC: *NAP1L1, NOL3, TPH1, RAF1, RSF1, ARAF, KRAS, MORF4L2, MKI67, ZFHX3, TECPR2, PQBP1, RNF41, SMARCD3, COMMD9, BRAF*

Apoptome = apoptosis; Epigenome = epigenetic regulation, Fibrosome = fibrosis; Growth Factor Signalome = growth factor signaling pathways; Inflammasome = immune and inflammatory responses; Metabolome = metabolomic regulation; Metastasome = metastatic regulation; NEDome = neuroendocrine differentiation; Neurome = neural gene regulation; Plurome = pluripotency; Proliferome = proliferative regulation; Secretome = secretory regulation; SSTRome = somatostatin receptor expression; Tfome = transcription factors; ROC = genes involved in tumor progression.

The approaches utilized for Algorithm #1 development are included in **Supplementary Figures S2** and **S3**. Subject numbers and break-down into the 80% training set and the 20% held-out testing set is included in **S2**. The best-performing model selection strategy based on house-keeping gene normalization and algorithm evaluation is included in **S3.**


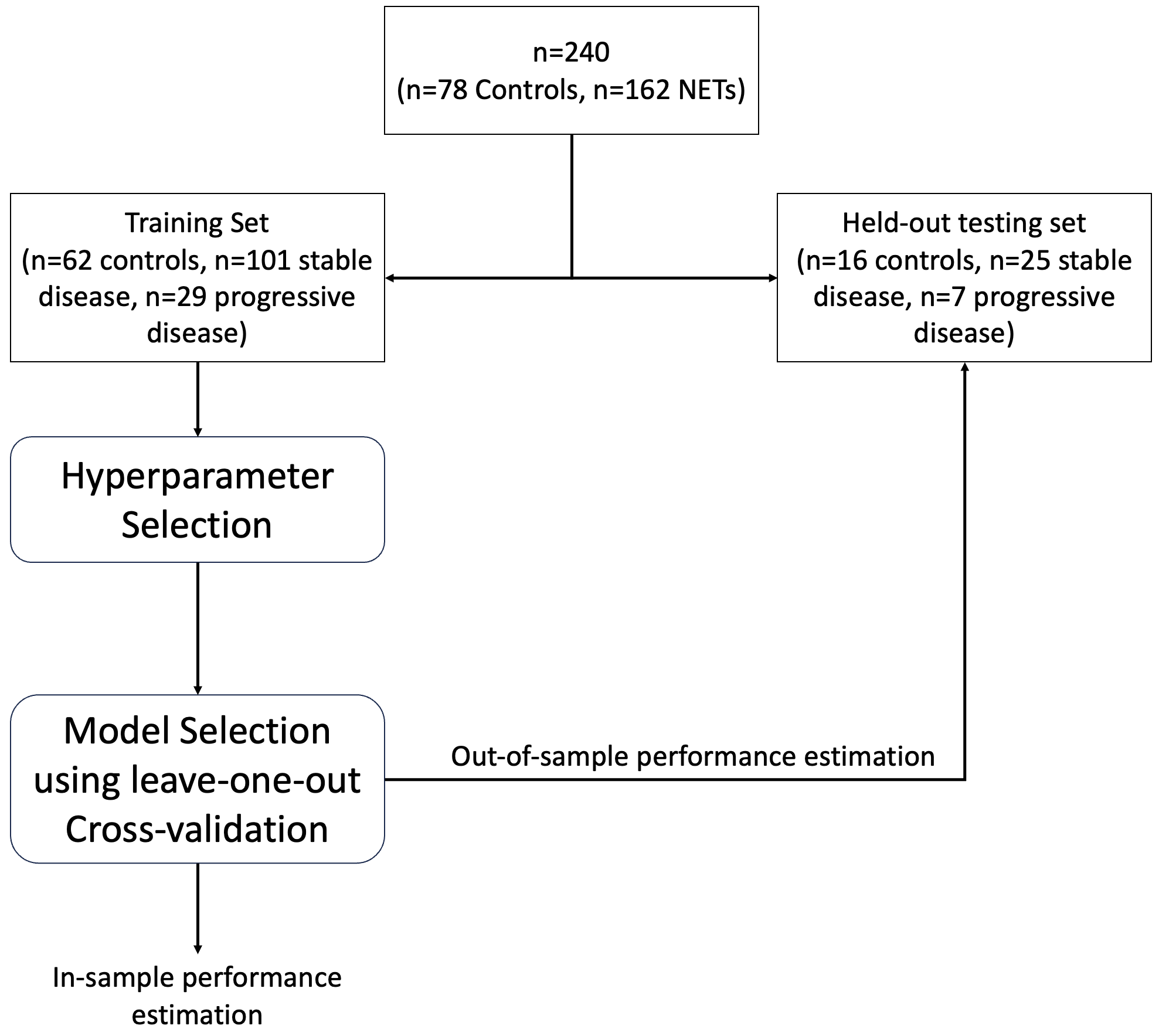


**Supplementary Figure S2. Flow chart demonstrating the training and hold-out testing set selection strategy and utilization (Training – USA).** Eighty percent of samples are used for hyperparameter and model selection using a leave-one-out cross validation approach. Twenty percent of samples are kept for hold-out testing (to evaluate algorithm performance).


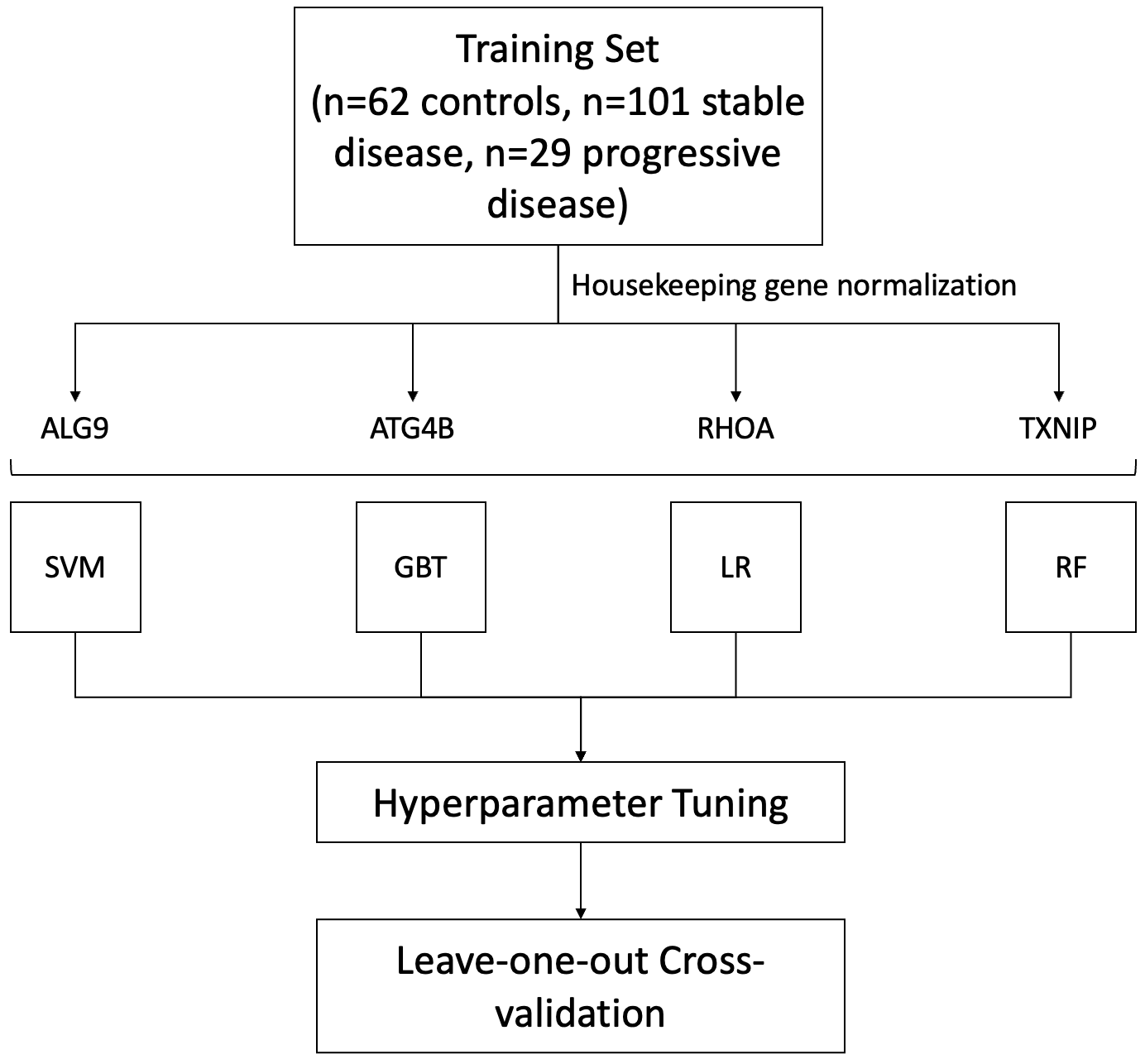


**Supplementary Figure S3. Flow chart demonstrating the best-performing model selection strategy.** All 51 target genes were normalized to each of the 4 housekeeping genes. Each normalized cohort was then evaluated using all 4 classifier approaches (SVM, GBT, LR and RF). A total of 16 (4 different housekeeping normalization sets x4 different classifiers) evaluations were undertaken on the target gene set.

SVM = support vector machine; GBT = gradient boosting technology; LR = linear regression; RF = random forest

The approaches utilized for Algorithm #2 development (USA – Training) are included in **Supplementary Figures S4** and **S5**. Subject numbers and break-down into the 80% training set and the 20% held-out testing set is included in **S4**. The best-performing model selection strategy based on house-keeping gene normalization and algorithm evaluation is included in **S5.**


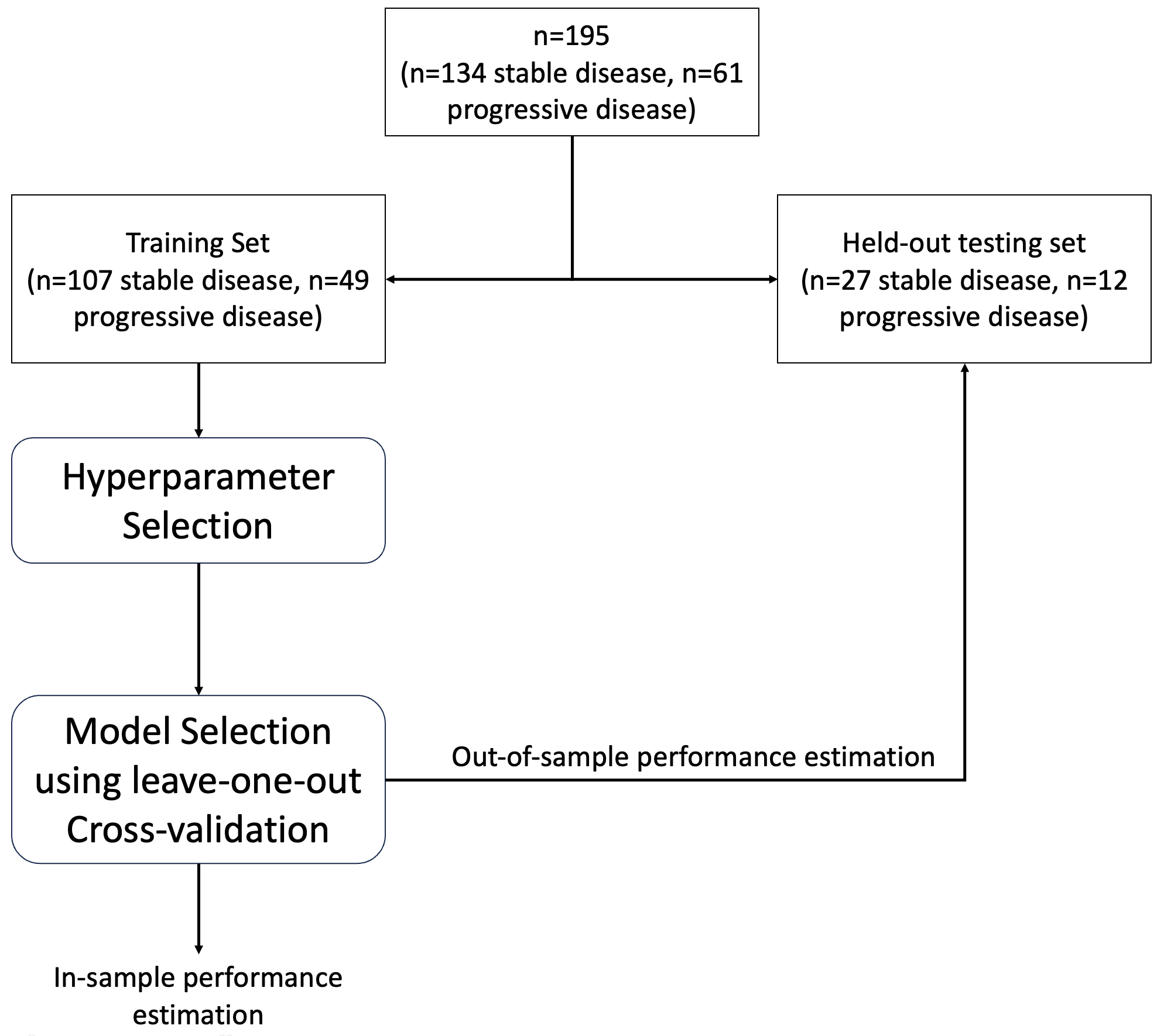


**Supplementary Figure S4. Flow chart demonstrating the training and hold-out testing set selection strategy and utilization.** Eighty percent of samples are used for hyperparameter and model selection using a leave-one-out cross validation approach. Twenty percent of samples are kept for hold-out testing (to evaluate algorithm performance).


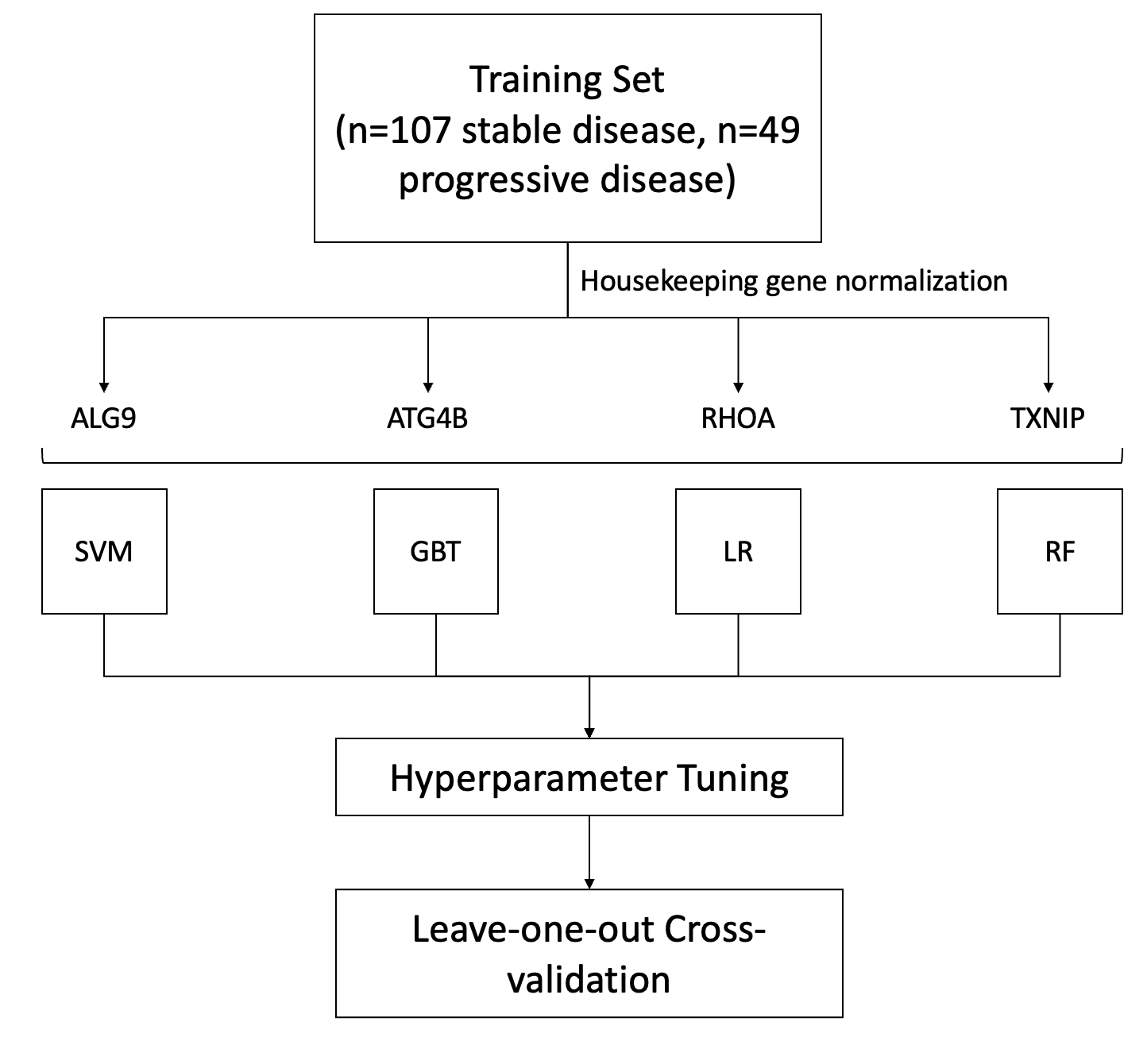


**Supplementary Figure S5. Flow chart demonstrating the best-performing model selection strategy.** All 51 target genes were normalized to each of the 4 housekeeping genes. Each normalized cohort was then evaluated using all 4 classifier approaches (SVM, GBT, LR and RF). A total of 16 (4 different housekeeping normalization sets x4 different classifiers) evaluations were undertaken on the target gene set.

SVM = support vector machine; GBT = gradient boosting technology; LR = linear regression; RF = random forest

**Assay Validation**

**Validation Sets**

Two different validation cohorts were examined (**Supplementary Figure S1** for blood sample collection, overview of demographics).

*Algorithm Validation Dataset (I):* Gene expression was evaluated in blood samples from 463 subjects including 186 healthy volunteers and 277 NET patients (**Supplementary Table S4**).

**Supplementary Table S4.** Demographics of Algorithm Validation Dataset I (USA / global (*n*=463)

|  | **Controls (*n*=186)** | **NET cohort**  **(*n*=277)** | | | ***p*-value** |
| --- | --- | --- | --- | --- | --- |
| **Age**  **Median (range)** | 55 (19-83) | 59 (20-87) | | | <0.0001* |
| **Gender (M:F)** | 117:69 | 141:136 | | | 0.013^†^ |
| **Ethnicity**  **A:B:H:W** | 13:36:34:103 | 50:13:22:192 | | | <0.0001^‡^ |
|  |  |  | | |  |
| **Location** |  | Lung:  Stomach:  Pancreas:  Small Bowel:  Appendix:  Colorectum:  CUP: | 22  9  113  86  9  17  21 | 7.9%  3.2%  40.8%  31.1%  3.2%  6.1%  7.7% | N/A |
| **Grade** |  | G1:  G2:  G3:  ND: | 110  78  15  74 | 39.7%  28.2%  5.4%  26.7% | N/A |
| **Stage** |  | Localized:  Metastatic:  ND: | 98  128  51 | 35.4%  46.2%  18.4% | N/A |
| **Clinical Status** |  | Stable:  Progressive:  ND: | 192  63  22 | 69.3%  22.7%  8.0% | N/A |

*Mann-Whitney U-test (2-tailed); ^†^Fisher’s exact test; ^‡^Chi^2^ test.

A=Asian, B=Black, H=Hispanic, W=White.

CUP = carcinoid of unknown primary; ND = no data

*129 (46.6%) were in monitoring/surveillance, 128 (46.2%) were on an SSA, 4 (1.4%) were being treated with chemotherapy (CAPTEM), 3 (1.1%) were on CAPTEM+SSA, 3 (1.1%) were on chemotherapy, 3 (1.1%) on Sutent, 1 (0.4%) was on Lacrotectinib (a TKI), and 6 (2.2%) had no information. No patient was treatment-naïve.*

*Algorithm Validation Dataset (II):* Gene expression was evaluated in blood samples from 291 NET patients (**Supplementary Table S5**).

**Supplementary Table S5.** Demographics of Algorithm Validation Dataset II (Switzerland) (*n*=291)

|  | **NET cohort**  **(*n*=291)** | | |
| --- | --- | --- | --- |
| **Age**  **Median (range)** | 64 (21-87) | | |
| **Gender (M:F)** | 135:156 | | |
| **Ethnicity**  **A:B:H:W** | 0:0:0:291 | | |
|  |  | | |
| **Location** | Lung:  Stomach:  Pancreas:  Small Bowel:  Appendix:  Colorectum:  CUP/other: | 47  12  87  102  8  9  26 | 16.2%  4.1%  29.9%  35.1%  2.7%  3.1%  8.9% |
| **Grade** | G1:  G2:  G3:  ND: | 116  136  26  13 | 39.9%  46.7%  8.9%  4.5% |
| **Stage** | Localized:  Metastatic: | 93  198 | 32.0%  68.0% |
| **Clinical Status** | Stable:  Progressive: | 181  110 | 62.2%  37.8% |

*135 (46%) were in monitoring/surveillance, 112 (38.5%) were on an SSA, 26 (9%) were being treated with chemotherapy (CAPTEM), 8 (3%) were on an SSA+Everolimus, 5 (1.7%) were on an SSA + Sutent, 3 (1%) were on PRRT, 1 was on everolimus and 1 on Sutent. No patient was treatment-naïve.*

*Algorithm Evaluation Datasets (I-III):* Gene expression was evaluated in blood samples from 147 patients with other malignancies, 19 patients with small cell lung cancer (SCLC) and 50 subjects with interstitial pulmonary fibrosis (IPF) (**Supplementary Table S6**).

**Supplementary Table S6.** Demographics of Evaluation Cohorts for Algorithm #1

|  | **Evaluation**  **Cohort**  **#I** | | | **Evaluation Cohort**  **#II** | **Evaluation Cohort**  **#III** |
| --- | --- | --- | --- | --- | --- |
|  | **Other malignancies**  **(*n*=147)** | | | **SCLC**  **(*n*=19)** | **IPF**  **(*n*=50)** |
| **Age**  **Median (range)** | 64 (27-88) | | | 62 (31-81) | 67 (43-87) |
| **Gender (M:F)** | 78:69 | | | 6:13 | 40:10 |
| **Ethnicity**  **A:B:H:W** | 4:8:16:119 | | | 0:0:0:50 | 0:0:0:50 |
|  |  | | |  |  |
| **Location** | Lung-AC:  Lung-SCC  Esophagus:  Stomach:  Pancreas:  Appendix  Colon:  GIST:  Kidney: | 53  6  7  6  23  1  47  1  3 | 36.0%  4.1%  4.8%  4.1%  15.6%  0.7%  32.0%  0.7%  2.0% |  |  |
| **Stage** | Localized:  Metastatic:  ND: | 51  70  26 | 34.7%  47.6%  17.7% |  |  |
| **Current treatment** | No:  Yes:  ND: | 80  65  2 | 54.4%  44.2%  1.4% |  |  |
| **Clinical Status** | Stable:  Progressive:  NED:  New diagnosis: | 55  59  6  27 | 37.4%  40.1%  4.1%  18.4% |  |  |

A=Asian, B=Black, H=Hispanic, W=White.

GIST = gastrointestinal stromal tumor; ND = no data

**RESULTS**

**Supplementary Table S7. Control vs. NET model performance metrics on the in-sample dataset (80% of samples: USA – Training).** Where appropriate, 95% CI ranges are shown below the performance statistics.

| **Classifier** | **House-keeping gene** | **Sensitivity** | **Specificity** | **PPV** | **AUROC** |
| --- | --- | --- | --- | --- | --- |
| SVM | *ALG9* | 0.94  (0.93-0.95) | 0.69  (0.68-0.70) | 0.86  (0.86-0.88) | 0.902  (0.901-0.903) |
|  | *ATG4B* | 0.94  (0.93-0.95) | 0.69  (0.68-0.70) | 0.86  (0.86-0.88) | 0.902  (0.901-0.903) |
|  | *RHOA* | 0.93  (0.92-0.94) | 0.69  (0.68-0.70) | 0.86  (0.86-0.88) | 0.902  (0.900 – 0.902) |
|  | *TXNIP* | 0.94  (0.93-0.95) | 0.69  (0.68-0.70) | 0.86  (0.86-0.88) | 0.903  (0.901-0.903) |
| GBT | *ALG9* | 0.94  (0.93-0.95) | 0.74  (0.73-0.75) | 0.88  (0.87-0.89) | 0.897  (0.895-0.897) |
|  | *ATG4B* | 0.94  (0.93-0.95) | 0.76  (0.75-0.77) | 0.89  (0.89-0.91) | 0.898  (0.895-0.898) |
|  | *RHOA* | 0.95  (0.94-0.96) | 0.76  (0.75-0.77) | 0.89  (0.89-0.91) | 0.898  (0.896-0.899) |
|  | *TXNIP* | 0.94  (0.93-0.95) | 0.79  (0.78-0.80) | 0.90  (0.89-0.81) | 0.898  (0.896-0.899) |
| LR | *ALG9* | 0.92  (0.91-0.93) | 0.68  (0.67-0.69) | 0.85  (0.84-0.86) | 0.881  (0.879-0.881) |
|  | *ATG4B* | 0.92  (0.91-0.93) | 0.68  (0.67-0.69) | 0.86  (0.85-0.87) | 0.880  (0.878-0.881) |
|  | *RHOA* | 0.92  (0.91-0.93) | 0.68  (0.67-0.69) | 0.86  (0.85-0.87) | 0.880  (0.878-0.881) |
|  | *TXNIP* | 0.92  (0.91-0.93) | 0.68  (0.67-0.69) | 0.86  (0.85-0.87) | 0.879  (0.877-0.879) |
| RF | *ALG9* | 0.96  (0.95-0.97) | 0.71  (0.70-0.72) | 0.87  (0.86-0.88) | 0.898  (0.896-0.900) |
|  | *ATG4B* | 0.96  (0.95-0.97) | 0.69  (0.69-0.71) | 0.87  (0.86-0.88) | 0.901  (0.899-0.902) |
|  | *RHOA* | 0.97  (0.96-0.98) | 0.69  (0.69-0.71) | 0.87  (0.86-0.88) | 0.900  (0.898-0.902) |
|  | *TXNIP* | 0.97  (0.96-0.98) | 0.69  (0.69-0.71) | 0.87  (0.86-0.88) | 0.900  (0.898-0.901) |


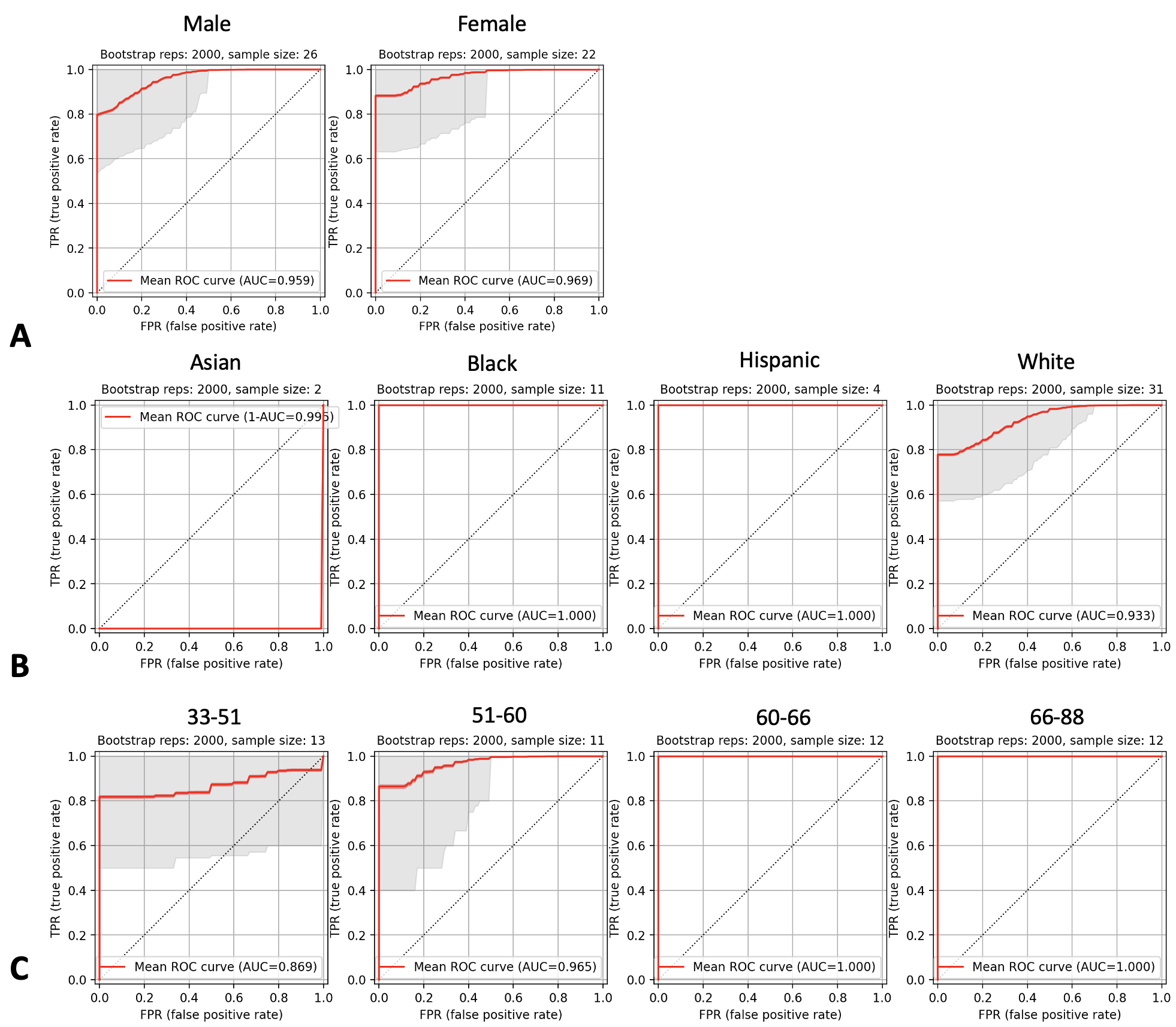


**Supplementary Figure S6. Receiver Operating Characteristic curves of a Random Forest model performance stratified by Sex (Male Female), Ethnicity (Asian, Black, Hispanic, White), and Age quartiles**. Figure rows correspond to model performance in each subgroup. Grey shaded areas correspond to 95% Confidence Intervals obtained using n=2000 bootstrap resamples. Sample sizes in figure titles reflect the number of samples within each subgroup that have non-empty demographic attribute.

**Supplementary Table S8. Control vs. NET model subgroup analysis on the out-of-sample testing set (20% of samples from USA – Training).** Sensitivities, Specificities, and PPVs were calculated using a model operating point of 0.50. Where appropriate, 95% CI ranges are shown below the performance statistics.

|  |  | **Sensitivity** | **Specificity** | **PPV** | **AUROC** |
| --- | --- | --- | --- | --- | --- |
|  |  |  |  |  |  |
| **Gender** | *Male* (n_NETs_=16) | 1.0  (0.99-1.00) | 0.4  (0.39-0.41) | 0.73  (0.72-0.74) | 0.959  (0.957-0.960) |
|  | *Female* (n_NETs_=16) | 1.0  (0.99-1.00) | 0.83  (0.82-0.84) | 0.94  (0.93-0.95) | 0.967  (0.965-0.969) |
|  |  |  |  |  |  |
| **Ethnicity** | Asian (n_NETs_=2) | 1.0 | 0.0 | 1.0 | N/A |
|  | Black (n_NETs_=7) | 1.00  (0.99-1.00) | 0.75  (0.74-0.76) | 0.88  (0.87-0.89) | 1.00  (0.99-1.00) |
|  | Hispanic (n_NETs_=2) | 1.00  (0.99-1.00) | 1.00  (0.99-1.00) | 1.00  (0.99-1.00) | 1.00  (0.99-10.100) |
|  | White (n_NETs_=21) | 1.00  (0.99-1.00) | 0.4  (0.39-0.41) | 0.78  (0.77-0.79) | 0.93  (0.931-0.934) |
|  |  |  |  |  |  |
| **Age** | *33-51* (n_NETs_=10) | 1.00  (0.99-1.00) | 0.00 | 0.77  (0.76-0.79) | 0.869  (0.863-0.873) |
|  | *51-60* (n_NETs_=5) | 1.00  (0.99-1.00) | 0.67  (0.66-0.68) | 0.71  (0.70-0.72) | 0.965  (0.962-0.967) |
|  | *60-66* (n_NETs_=7) | 1.00  (0.99-1.00) | 0.80  (0.79-0.81) | 0.88  (0.87-0.89) | 1.00  (0.99-1.00) |
|  | *66-88* (n_NETs_=10) | 1.00  (0.99-1.00) | 0.5  (0.49-0.51) | 0.91  (0.90-0.92) | 1.00  (0.99-1.00) |

**Supplementary Table S9. Stable vs. Progressive model performance metrics on the in-sample dataset (80% samples: USA – Training).** Where appropriate, 95% CI ranges are shown below the performance statistics.

| **Classifier** | **House-keeping gene** | **Sensitivity** | **Specificity** | **PPV** | **AUROC** |
| --- | --- | --- | --- | --- | --- |
| SVM | *ALG9* | 0.47  (0.46-0.48) | 0.99  (0.98-1.00) | 0.96  (0.95-0.97) | 0.773  (0.769-0.773) |
|  | *ATG4B* | 0.47  (0.46-0.48) | 0.99  (0.98-1.00) | 0.96  (0.95-0.97) | 0.768  (0.764-0.769) |
|  | *RHOA* | 0.47  (0.46-0.48) | 0.99  (0.98-1.00) | 0.96  (0.95-0.97) | 0.769  (0.765 – 0.769) |
|  | *TXNIP* | 0.47  (0.46-0.48) | 0.99  (0.98-1.00) | 0.96  (0.95-0.97) | 0.769  (0.765-0.770) |
| GBT | *ALG9* | 0.67  (0.66-0.68) | 0.95  (0.94-0.96) | 0.87  (0.86-0.88) | 0.803  (0.801-0.805) |
|  | *ATG4B* | 0.67  (0.66-0.68) | 0.92  (0.91-0.93) | 0.79  (0.78-0.80) | 0.757  (0.754-0.759) |
|  | *RHOA* | 0.67  (0.66-0.68) | 0.95  (0.94-0.96) | 0.87  (0.86-0.88) | 0.802  (0.797-0.801) |
|  | *TXNIP* | 0.67  (0.66-0.68) | 0.95  (0.94-0.96) | 0.87  (0.86-0.88) | 0.804  (0.800-0.805) |
| LR | *ALG9* | 0.34  (0.33-0.35) | 1.00  (0.99-1.00) | 1.00  (0.99-1.00) | 0.677  (0.674-0.678) |
|  | *ATG4B* | 0.34  (0.33-0.35) | 1.00  (0.99-1.00) | 1.00  (0.99-1.00) | 0.67  (0.676-0.681) |
|  | *RHOA* | 0.34  (0.33-0.35) | 1.00  (0.99-1.00) | 1.00  (0.99-1.00) | 0.677  (0.673-0.678) |
|  | *TXNIP* | 0.34  (0.33-0.35) | 1.00  (0.99-1.00) | 1.00  (0.99-1.00) | 0.678  (0.675-0.680) |
| RF | *ALG9* | 0.65  (0.64-0.66) | 0.96  (0.95-0.97) | 0.89  (0.88-0.90) | 0.809  (0.805-0.809) |
|  | *ATG4B* | 0.65  (0.64-0.66) | 0.96  (0.95-0.97) | 0.89  (0.88-0.90) | 0.808  (0.804-0.808) |
|  | *RHOA* | 0.51  (0.50-0.52) | 0.98  (0.97-0.99) | 0.93  (0.92-0.94) | 0.754  (0.749-0.754) |
|  | *TXNIP* | 0.49  (0.48-0.50) | 0.98  (0.97-0.99) | 0.92  (0.91-0.93) | 0.766  (0.761-0.766) |


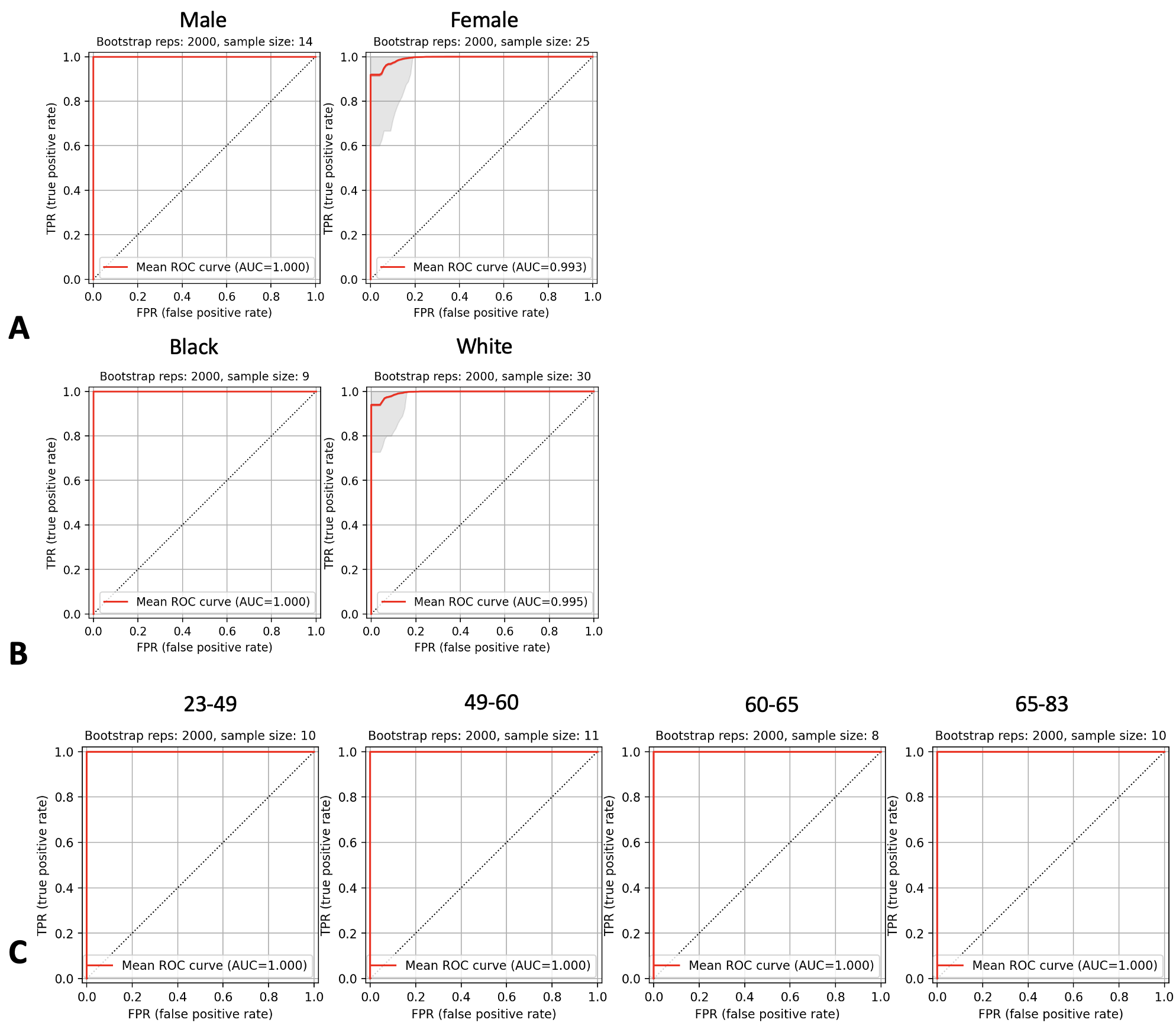


**Supplementary Figure S7. Receiver Operating Characteristic curves of a Random Forest model performance stratified by Sex (Male Female), Ethnicity (Black, White), and Age quartiles**. Figure rows correspond to model performance in each subgroup. Grey shaded areas correspond to 95% Confidence Intervals obtained using n=2000 bootstrap resamples. Sample sizes in figure titles reflect the number of samples within each subgroup that have non-empty demographic attribute.

**Supplementary Table S10. Stable vs. Progressive NET model subgroup analysis on the out-of-sample testing set (20% of samples: USA – Training).** Sensitivities, Specificities, and PPVs were calculated using a model operating point of 0.50. Where appropriate, 95% CI ranges are shown below the performance statistics.

|  |  | **Sensitivity** | **Specificity** | **PPV** | **AUROC** |
| --- | --- | --- | --- | --- | --- |
|  |  |  |  |  |  |
| **Gender** | *Male* (n_Progressive_=4) | 1.0  (0.99-1.00) | 0.90  (0.89-0.91) | 0.80  (0.78-0.81) | 1.00  (0.999-1.00) |
|  | *Female* (n_Progressive_=8) | 0.75  (0.73-0.77) | 1.0  (0.99-1.00) | 1.0  (0.99-1.00) | 0.993  (0.992-1.00) |
|  |  |  |  |  |  |
| **Ethnicity** | Black (n_Progressive_=1) | 1.0  (0.99-1.00) | 0.00 | 0.00 | 1.00  (0.99-1.00) |
|  | White (n_Progressive_=11) | 0.91  (0.89-0.93) | 0.95  (0.94-0.0.97) | 0.91  (0.90-0.93) | 0.995  (0.994-0.995) |
|  |  |  |  |  |  |
| **Age** | *23-49* (n_Progressive_ =3) | 1.00  (0.99-1.00) | 0.86  (0.85-0.88) | 0.75  (0.74-0.77) | 1.00  (0.99-1.00) |
|  | *49-60* (n_Progressive_ =3) | 0.67  (0.66-0.68) | 1.00  (0.99-1.00) | 1.00  (0.99-1.00) | 1.00  (0.99-1.00) |
|  | *60-65* (n_Progressive_ =3) | 0.67  (0.66-0.68) | 1.00  (0.99-1.00) | 1.00  (0.99-1.00) | 1.00  (0.99-1.00) |
|  | *65-83* (n_Progressive_ =3) | 1.00  (0.99-1.00) | 1.00  (0.99-1.00) | 1.00  (0.99-1.00) | 1.00  (0.99-1.00) |

NETest scores (Algorithm #1) in the different evaluation cohorts are included in **Supplementary Table S11.**

**Supplementary Table S11.** NETest positivity in the Evaluation Cohorts

| **Other malignancies**  **(*n*=147)** | | | | | | | | | **SCLC**  **(n=19)** | **IPF**  **(n=50)** |
| --- | --- | --- | --- | --- | --- | --- | --- | --- | --- | --- |
| **GI and Pancreatic cancers**  (*n*=84) | | | | | **Others**  (*n*=4) | | **Lung**  (*n*=59) | |  |  |
| **Esophagus**  (*n*=7) | **Gastric**  (*n*=6) | **Pancreas**  (*n*=23) | **Appendix***  *(n*=1*)* | **Colon**  (*n*=47) | **Kidney**  (*n*=3) | **GIST**  (*n*=1) | **LUAD**  (*n*=53) | **SCC**  (*n*=6) |  |  |
| 1  (14.3%) | 1  (16.7%) | 1  (4.3%) | 1  (100%) | 3  (6.4%) | 3  (100%) | 0  (0%) | 4  (7.5%) | 1  (16.7%) | 18  (94.7%) | 0  (0%) |

*Mucinous adenocarcinoma

LUAD = lung adenocarcinoma; SCC = squamous cell cancers


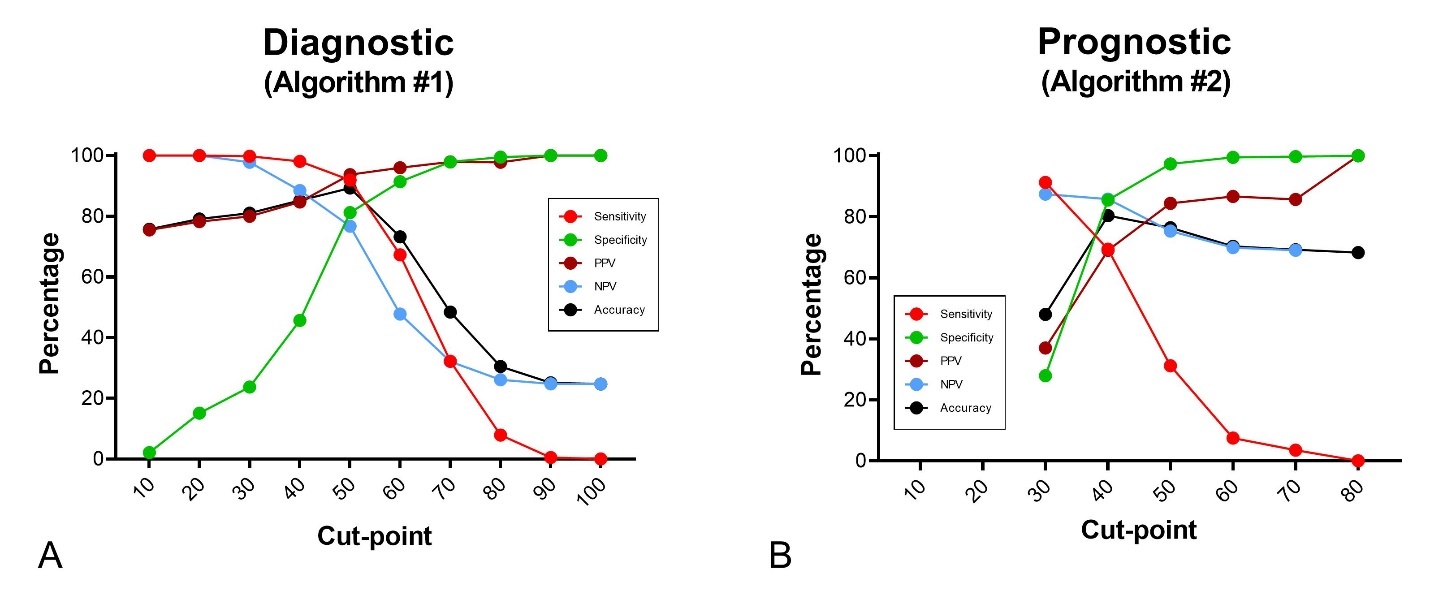


**Supplementary Figure S8.** Metrics evaluating different cut-points. The metrics for each of the two algorithms (diagnostic and prognostic) are included below. The optimal cut-point for Algorithm #1 (Diagnostic) in the Validation Cohort I + II (*n*=186 controls, *n*=568 NETs) is 50. The optimal cut-point for Algorithm #2 (Prognostic) in Validation Cohort I + II (*n*=373 SD, *n*=163 PD) is 40.

SD = stable disease, PD = progressive disease

**Supplementary Table S12. Cut-point evaluations for Algorithm #1: NETest Diagnostic**. Sensitivity, specificity, positive predictive value (PPV), negative predictive value (NPV) and accuracy calculations are tabulated. Data is calculated from the Validation Cohort I + II (*n*=186 controls, *n*=568 NETs) and is graphed in **Figure S8A**. The information from cut-point 50 is highlighted.

| **Cut-point (%)** | **Sensitivity** | **Specificity** | **PPV** | **NPV** | **Accuracy** |
| --- | --- | --- | --- | --- | --- |
| 10 | 100 (99.3-100) | 2.1 (0.6-5.4) | 75.5 (75.1-75.9) | 100 (39.8-100) | 75.7 (72.4-78.7) |
| 20 | 100 (99.4-100) | 15.1 (10.2-21) | 78.2 (77.2-79.3) | 100 (87.7-100) | 79.1 (76.0-81.9) |
| 30 | 99.8 (99.0-100) | 23.7 (17.8-30.4) | 80 (78.7-81.2) | 97.8 (85.9-99.7) | 81.0 (78.1-83.8) |
| 40 | 98.1 (96.6-99.0) | 45.7 (38.4-53.2) | 84.7 (82.9-86.3) | 88.5 (80.8-93.4) | 85.2 (82.4-87.6) |
| 50 | 91.9 (89.4-94.0) | 81.2 (74.8-86.5) | 93.7 (91.7-95.3) | 76.7 (71.2-81.4) | 89.3 (86.8-91.4) |
| 60 | 67.3 (63.2-71.1) | 91.4 (86.4-95) | 96.0 (93.7-97.5) | 47.8 (44.6-50.9) | 73.2 (69.9-76.3) |
| 70 | 32.2 (28.4-36.2) | 97.9 (94.6-99.4) | 97.9 (94.5-99.2) | 32.1 (30.8-33.4) | 48.4 (47.8-52.0) |
| 80 | 7.9 (5.8-10.4) | 99.5 (97.0-100) | 97.8 (86.2-99.7) | 26.1 (25.6-26.6) | 30.5 (27.2-33.9) |
| 90 | 0.5 (0.1-1.5) | 100 (98-100) | 100 (29.2-100) | 24.8 (24.7-24.9) | 25.1 (22.0-28.3) |
| 100 | 0.0 (0.0-0.7) | 100 (98-100) | 100 (29.2-100) | 24.7 (24.7-24.7) | 24.7 (21.6-27.9) |

**Supplementary Table S13. Cut-point evaluations for Algorithm #2: NETest Prognostic.** Sensitivity, specificity, positive predictive value (PPV), negative predictive value (NPV) and accuracy calculations are tabulated. Data is calculated from Validation Cohort I + II (*n*=373 SD, *n*=163 PD) is 40. and is graphed in **Figure S8B**. The information from cut-point 40 is highlighted.

| **Cut-point (%)** | **Sensitivity** | **Specificity** | **PPV** | **NPV** | **Accuracy** |
| --- | --- | --- | --- | --- | --- |
| 10 |  |  |  |  |  |
| 20 |  |  |  |  |  |
| 30 | 91.3 (86.1-95.1) | 27.9 (23.4-32.7) | 37.0 (35.2-38.8) | 87.4 (80.6-92.0) | 48.0 (43.7-52.3) |
| 40 | 69.4 (61.9-76.1) | 85.5 (81.5-88.9) | 69.0 (6..0-73.4) | 85.8 (82.7-88.3) | 80.4 (76.8-83.7) |
| 50 | 31.2 (24.4-38.7) | 97.3 (95.1-98.7) | 84.4 (73.8-91.2) | 75.3 (73.4-77.2) | 76.4 (72.6-79.9) |
| 60 | 7.5 (4.1-12.5) | 99.5 (98.1-99.90 | 86.7 (59.7-96.6) | 69.9 (69.0-70.8) | 70.3 (66.3-74.1) |
| 70 | 3.5 (1.3-7.4) | 99.7 (98.5-100) | 85.7 (42.1-98.0) | 69. (68.4-69.6) | 69.2 (65.2-73.1) |
| 80 | 0.0 (0.0-2.1) | 100 (99.0-100) |  | 68.3 (68.3-68.3) | 68.3 (68.3-68.3) |
| 90 |  |  |  |  |  |
| 100 |  |  |  |  |  |

**Supplementary Table S14. Control vs. NET model subgroup analysis on the combined Validation Cohort (Set I+II: *n*=754).** Sensitivities, Specificities, and PPVs were calculated using a model operating point of 0.50. 95% CI ranges are shown below the performance statistics.

|  |  | **Sensitivity** | **Specificity** | **PPV** | **AUROC** |
| --- | --- | --- | --- | --- | --- |
|  |  |  |  |  |  |
| **Gender** | *Male*  (n_NETs_=276; n_CON_=117) | 0.92  (0.88-0.95) | 0.78  (0.70-0.86) | 0.91  (0.88-0.94) | 0.667  (0.618-0.714) |
|  | *Female*  (n_NETs_=292; n_CON_=69) | 0.92  (0.88-0.95) | 0.86  (0.75-0.93) | 0.96  (0.94-0.98) | 0.936  (0.905-0.959) |
|  |  |  |  |  |  |
| **Ethnicity** | Asian  (n_NETs_=54; n_CON_=13) | 0.89  (0.77-0.96) | 0.92  (0.64-1.0) | 0.98  (0.88-1.0) | 0.945  (0.861-0.986) |
|  | Black  (n_NETs_=21; n_CON_=36) | 1.00  (0.4-1.00) | 0.69  (0.52-0.84) | 0.66  (0.54-0.76) | 0.893  (0.782-0.959) |
|  | Hispanic (n_NETs_=33; n_CON_=34) | 0.88  (0.72-0.97) | 0.77  (0.59-0.89) | 0.78  (0.66-0.87) | 0.864  (0.758-0.936) |
|  | White  (n_NETs_=460; n_CON_=103) | 0.92  (0.89-0.95) | 0.85  (0.77-0.92) | 0.97  (0.95-0.98) | 0.932  (0.909-0.952) |
|  |  |  |  |  |  |
| **Age*** | *19-52*  (n_NETs_=137; n_CON_=65) | 0.96  (0.91-0.98) | 0.86  (0.75-0.94) | 0.94  (0.89-0.96) | 0.952  (0.912-0.977) |
|  | *53-60*  (n_NETs_=114; n_CON_=69) | 0.87  (0.80-0.92) | 0.87  (0.76-0.94) | 0.93  (0.87-0.96) | 0.886  (0.831-0.928) |
|  | *61-69*  (n_NETs_=145; n_CON_=41) | 0.90  (0.84-0.94) | 0.78  (0.62-0.89) | 0.94  (0.89-0.96) | 0.908  (0.857-0.946) |
|  | *70-87*  (n_NETs_=172; n_CON_=11) | 0.90  (0.85-0.94) | 0.91  (0.59-1.00) | 0.99  (0.96-1.00) | 0.934  (0.888-0.965) |

*Age based on quartiles

**Supplementary Table S15. Stable vs. Progressive model subgroup analysis on the combined Validation Cohort (Set I+II: *n*=536).** Sensitivities, Specificities, and PPVs were calculated using a model operating point of 0.40. 95% CI ranges are included below the performance statistics.

|  |  | **Sensitivity** | **Specificity** | **PPV** | **AUROC** |
| --- | --- | --- | --- | --- | --- |
| **Gender** | *Male*  (n_Progressive_=84;  N_Stable_=180) | 0.68  (0.57-0.78) | 0.86  (0.80-0.91) | 0.70  (0.61-0.77) | 0.802  (0.748-0.848) |
|  | *Female* (n_Progressive_=89;  N_Stable_=193) | 0.71  (0.60-0.80) | 0.85  (0.79-0.90) | 0.69  (0.60-0.76) | 0.805  (0.754-0.850) |
|  |  |  |  |  |  |
| **Ethnicity** | Asian  (n_Progressive_=18;  N_Stable_=35) | 0.50  (0.26-0.74) | 0.63  (0.45-0.79) | 0.41  (0.27-0.57) | 0.606  (0.462-0.737) |
|  | Black  (n_Progressive_=4;  N_Stable_=14) | 0.75  (0.19-0.99) | 0.86  (0.57-0.98) | 0.60  (0.27-0.86) | 0.830  (0.582-0.963) |
|  | Hispanic (n_Progressive_=8;  N_Stable_=21) | 0.50  (0.16-0.84) | 0.76  (0.53-0.92) | 0.44  (0.22-0.69) | 0.795  (0.604-0.921) |
|  | White  (n_Progressive_=143;  N_Stable_=303) | 0.71  (0.63-0.79) | 0.89  (0.85-0.92) | 0.75  (0.68-0.81s) | 0.826  (0.788-0.860) |
|  |  |  |  |  |  |
| **Age*** | *20-53*  (n_Progressive_=36;  N_Stable_=110) | 0.72  (0.55-0.86) | 0.81  (0.72-0.88) | 0.55  (0.45-0.66) | 0.815  (0.742-0.874) |
|  | *54-62*  (n_Progressive_=45;  N_Stable_=85) | 0.73  (0.58-0.85) | 0.86  (0.77-0.93) | 0.73  (0.61-0.83) | 0.805  (0.726-0.869) |
|  | *63-72*  (n_Progressive_=49;  N_Stable_=100) | 0.63  (0.48-0.77) | 0.92  (0.85-0.97) | 0.80  (0.66-0.89) | 0.812  (0.739-0.871) |
|  | *73-87*  (n_Progressive_=43;  N_Stable_=78) | 0.70  (0.54-0.83) | 0.83  (0.73-0.91) | 0.70  (0.58-0.80) | 0.805  (0.724-0.872) |

*Age based on quartiles

**References**

1. Kidd M, Drozdov I, Modlin I. Blood and tissue neuroendocrine tumor gene cluster analysis correlate, define hallmarks and predict disease status. *Endocr Relat Cancer*. 2015; **22**(4): 561-75. doi: 10.1530/ERC-15-0092. Epub 2015 Jun 2.

2. Kidd M, Kitz A, Drozdov I, Modlin I. Neuroendocrine Tumor Omic Gene Cluster Analysis Amplifies the Prognostic Accuracy of the NETest. *Neuroendocrinology*. 2021; **111**(5): 490-504.

3. Laskaratos FM, Mandair D, Hall A, Alexander S, von Stempel C, Bretherton J, Luong T, Watkins J, Ogunbiyi O, Rombouts K, Caplin M, Toumpanakis C. Clinicopathological correlations of mesenteric fibrosis and evaluation of a novel biomarker for fibrosis detection in small bowel neuroendocrine neoplasms. *Endocrine*. 2019; **9**(10): 019-02107.
